# Macrophage Coordination of the Interferon Lambda Immune Response

**DOI:** 10.3389/fimmu.2019.02674

**Published:** 2019-11-19

**Authors:** Scott A. Read, Ratna Wijaya, Mehdi Ramezani-Moghadam, Enoch Tay, Steve Schibeci, Christopher Liddle, Vincent W. T. Lam, Lawrence Yuen, Mark W. Douglas, David Booth, Jacob George, Golo Ahlenstiel

**Affiliations:** ^1^Blacktown Medical School, Western Sydney University, Blacktown, NSW, Australia; ^2^Storr Liver Centre, The Westmead Institute for Medical Research, The University of Sydney and Westmead Hospital, Westmead, NSW, Australia; ^3^Centre for Immunology and Allergy Research, The Westmead Institute for Medical Research, The University of Sydney and Westmead Hospital, Westmead, NSW, Australia; ^4^Department of Upper Gastrointestinal Surgery, Westmead Hospital, Westmead, NSW, Australia; ^5^Discipline of Surgery, University of Sydney, Sydney, NSW, Australia; ^6^Centre for Infectious Diseases and Microbiology, Marie Bashir Institute for Infectious Diseases and Biosecurity, University of Sydney at Westmead Hospital, Westmead, NSW, Australia; ^7^Blacktown Hospital, Western Sydney Local Health District (WSLHD), Blacktown, NSW, Australia

**Keywords:** macrophage, interferon lambda, innate immunity, liver, Kupffer

## Abstract

Lambda interferons (IFN-λs) are a major component of the innate immune defense to viruses, bacteria, and fungi. In human liver, IFN-λ not only drives antiviral responses, but also promotes inflammation and fibrosis in viral and non-viral diseases. Here we demonstrate that macrophages are primary responders to IFN-λ, uniquely positioned to bridge the gap between IFN-λ producing cells and lymphocyte populations that are not intrinsically responsive to IFN-λ. While CD14^+^ monocytes do not express the IFN-λ receptor, IFNLR1, sensitivity is quickly gained upon differentiation to macrophages *in vitro*. IFN-λ stimulates macrophage cytotoxicity and phagocytosis as well as the secretion of pro-inflammatory cytokines and interferon stimulated genes that mediate immune cell chemotaxis and effector functions. In particular, IFN-λ induced CCR5 and CXCR3 chemokines, stimulating T and NK cell migration, as well as subsequent NK cell cytotoxicity. Using immunofluorescence and cell sorting techniques, we confirmed that human liver macrophages expressing CD14 and CD68 are highly responsive to IFN-λ *ex vivo*. Together, these data highlight a novel role for macrophages in shaping IFN-λ dependent immune responses both directly through pro-inflammatory activity and indirectly by recruiting and activating IFN-λ unresponsive lymphocytes.

## Introduction

Lambda interferons (IFNL and IFN-λ), also known as type III IFNs, are a family of cytokines comprising four members: IFN-λ1 (IL29), IFN-λ2 (IL28A), IFN-λ3 (IL28B), and IFN-λ4. While all IFN-λs signal through a unique IFNLR1:IL10Rβ receptor complex, they activate a gene signature similar to type I IFNs, IFN-α, and IFN-β (1). Both type I and III IFNs activate the transcription of hundreds of interferon stimulated genes (ISGs) (1) that exhibit numerous autocrine and paracrine antiviral roles. Although IFNs are required to clear most viral infections, prolonged expression due to environmental or genetic factors can stimulate sustained immune activation, driving tissue damage, and fibrosis (2, 3).

Elevated IFN-λ3 production has demonstrated a strong association with *IFNL* genotype and hepatic inflammation, increasing the risk of both viral (HBV and HCV) and non-viral (non-alcoholic steatohepatitis, NASH) related progressive liver inflammation and fibrosis (4). Furthermore, these effects appear to be independent of IFN-λ4 activity, suggesting that IFN-λ3 may be a primary mediator of inflammation (5). While the precise mechanisms remain uncertain, peripheral and hepatic immune cell populations vary according to the *IFNL* polymorphism in patients with chronic HCV infection, suggesting that IFN-λs can prompt immune cell migration to tissues (5, 6).

IFN-λ activity is restricted to specific tissues due to selective IFNLR1 expression. In humans, epithelial cells within the lung, intestine and liver among others, are uniquely IFN-λ sensitive. In particular, IFN-λs have been shown to protect against pulmonary influenza and human metapneumovirus (HMPV) (7, 8), gastrointestinal rotavirus and norovirus (9, 10) and hepatic HBV and HCV (11, 12). While the majority of human studies have been performed *in vitro*, murine studies have shown potent antiviral effects of IFN-λs against numerous viruses including influenza and SARS coronavirus (13, 14), rotavirus, norovirus, and reovirus (15–17). It should be noted that IFNLR1 expression may differ between humans and mice, as exemplified in murine hepatocytes that do not respond to IFN-λ (18). Immune cells also demonstrate very restricted IFN-λ responsiveness with myeloid immune cell populations harboring the strongest responses: Human dendritic cells (DC) and neutrophils are highly responsive to IFN-λs (19–23), whereas natural killer (NK) and T cells have consistently demonstrated minimal responsiveness (21, 24, 25). Investigation of monocyte and B cell responsiveness has produced conflicting results (21, 24, 26–30), perhaps confounded by studies utilizing co-culture models in the presence of IFN-λ responsive cells (31–33). As such, isolation of pure immune cell subsets is required to unequivocally define IFN-λ sensitivity.

Here, we demonstrate that human macrophages, not monocytes, are a dominant, physiologically relevant IFN-λ responsive population capable of orchestrating tissue inflammation. This is achieved through a direct immuno-stimulatory response to IFN-λ and subsequent NK and T cell chemotaxis and activation. *In vivo*, macrophages are responsive to IFN-λ3 and accumulate in inflamed human liver. These data suggest a novel role of macrophages as key players in modulating the IFN-λ response in acute infection, as well as chronic disease.

## Results

### Macrophages Not Monocytes Are Responsive to IFN-λ

**Figure 1:**
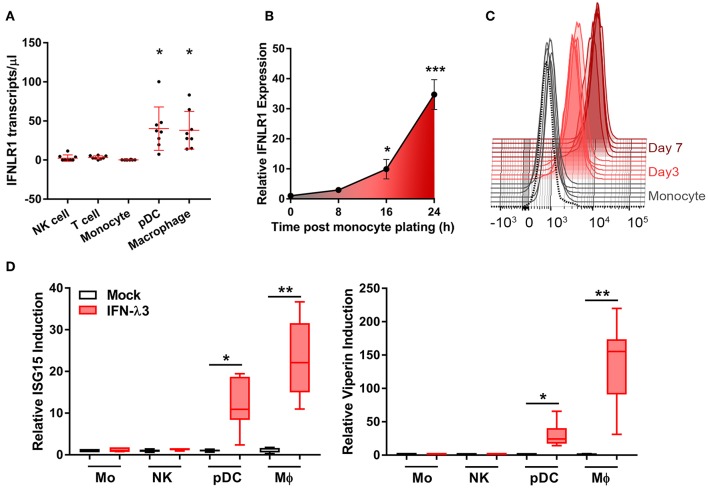
Macrophages but not monocytes are highly IFN-λ3 responsive. To investigate IFN-λ responsiveness, immune cell subsets were magnetically isolated and *IFNLR1* expression was quantified by ddPCR **(A)**. Mϕ and pDC *IFNLR1* expression was significantly higher than monocyte, NK and T cell populations (*p* < 0.05, *n* = 8). Time course analysis demonstrated that *IFNLR1* expression quickly rises following monocyte plating, reaching a 30× increase at 24 h even in the absence of GM-CSF addition (*p* < 0.001, *n* ≥ 5) **(B)**. Similarly, IFNLR1 surface expression during macrophage differentiation (MFI) increased at days 3 and 7 (*p* < 0.001, *n* ≥ 5) **(C)**. Isolated immune cell subsets were treated with 100 ng/ml IFN-λ3 for 8 h and the expression of ISGs *viperin* and *ISG15* were examined (*n* ≥ 7) **(D)**. Consistent with *IFNLR1* expression, Mϕs and pDCs were highly responsive to IFN-λ3, whereas monocytes and NK cells were not (*n* ≥ 5). Data are representative of two **(B,C)** and three independent experiments **(A,D)**. One-way ANOVA **(A)**, Mann–Whitney test **(B,D)**, paired *t*-test **(C)**, *p < 0.05, **p < 0.01, ***p < 0.001 (mean ± SE).

To address the uncertainty surrounding monocyte and macrophage (Mϕ) IFN-λ-responsiveness, we measured mRNA expression of the IFN-λ receptor, *IFNLR1*, in blood leukocytes by digital droplet PCR (ddPCR). DdPCR enables the precise quantification of RNA transcripts by performing the PCR reaction within >10,000 oil droplets, and calculating transcript copies using Poisson's law of small numbers (34). *IFNLR1* expression in freshly isolated monocytes and in Mϕs cultured for 7 days with GM-CSF was compared to IFN-λ responsive cells (pDCs) and “unresponsive” cells (NK and T cells). Similar to NK and T cells, monocytes expressed minimal *IFNLR1* transcript. Mϕ and pDC *IFNLR1* expression was significantly increased compared to other populations, suggesting IFN-λ responsiveness (Figure 1A). Increased abundance of *IFNLR1* was confirmed following monocyte to macrophage differentiation using seven datasets from the NCBI Gene Expression Omnibus (35) (Figure S1). To assess *IFNLR1* expression during differentiation, *IFNLR1* expression was quantified over 24 h (qPCR, no differentiation stimulus) and 7 days (flow cytometry, GM-CSF differentiation) following monocyte plating. Expression of the *IFNLR1* transcript was quickly increased as early as 16 h post-plating, reaching a 30-fold increase at 24 h (Figure 1B). IFNLR1 surface expression was significantly increased at day 3 (monocyte IFNLR1 MFI 927 vs. day 3 Mϕ 3199) and further increased at day 7 (day 7 Mϕ IFNLR1 MFI 10,412) (Figure 1C).

To test monocyte and Mϕ responsiveness to IFN-λ, cells isolated and cultured as in Figure 1A were treated with 100 ng/ml IFN-λ3 for 8 h. This concentration is not a saturation dose, but is high enough to evoke a strong interferon response in Mϕs (Figure S2). Consistent with increased *IFNLR1* expression, Mϕ and pDC mRNA expression of *viperin* and *ISG15* were markedly increased (Figure 1D), whereas monocytes and NK cells demonstrated negligible responses.

### Differentiation Method Regulates IFN-λ Responsiveness

Mϕ differentiation medium containing IFN-γ and LPS or interleukin 4 (IL-4) and IL-13 are often used to generate pro-inflammatory (M1) or anti-inflammatory (M2) Mϕs, respectively, but do not reflect the spectrum of macrophage activation *in vivo* (36). To avoid generating Mϕs whose IFN-λ sensitivity is influenced by phenotype skewing, monocytes were differentiated for 7 days with GM-CSF or M-CSF alone, as previously described (37, 38). The resulting Mϕ populations are differentially responsive to inflammatory stimuli, and are thus M1- or M2-shifted while maintaining some baseline characteristics of polarized Mϕs (Figure S3).When compared to monocyte derived DCs (MDDCs) generated using IL-4 and GM-CSF, the resulting Mϕs express elevated surface expression of CD14 and CD16, reduced CD1C, and unlike MDDCs, adhere strongly to culture dishes (Figure S4). M1- and M2-shifted Mϕs will be termed GM-Mϕs and M-Mϕs for the remainder of the manuscript.

**Figure 2:**
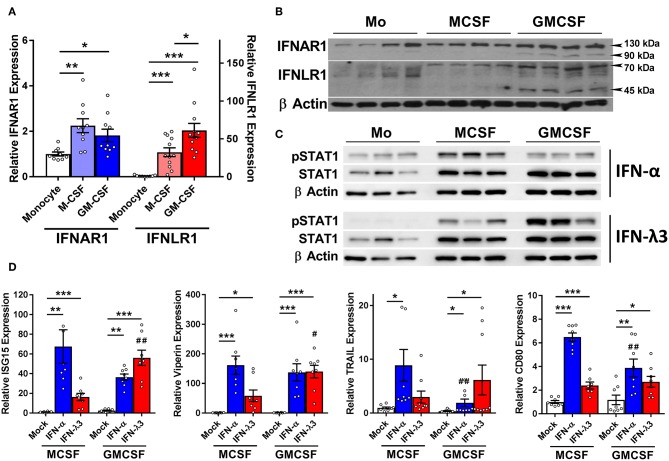
M-CSF and GM-CSF differentiated macrophages respond differently to IFN-α and IFN-λ3. Following M- and GM-CSF stimulated differentiation, IFN-λ responsiveness of Mϕ populations was examined, and compared to IFN-α. M-CSF and GM-CSF Mϕ subsets both increased the expression of *IFNAR1* ~2-fold following differentiation from monocytes, and *IFNLR1* transcripts ~30- and 60-fold, respectively **(A)** (*n* ≥ 9). Western blot of IFNAR1 and IFNLR1 from four healthy subjects confirmed these findings **(B)** (*n* = 7, total). Supporting these findings, phosphorylation of STAT1 was detected by Western blot in both monocytes and macrophages following 15 min of IFN-α treatment and macrophages only following IFN-λ3 **(C)**. ISG transcripts for *ISG15, viperin, CD80*, and *TRAIL* were examined to measure Mϕ sensitivity to IFN-α (50 U/ml) and IFN-λ3 (100 ng/ml) **(D)** (*n* = 8). M-CSF differentiated Mϕs were more responsive to IFN-α, whereas GM-CSF differentiated Mϕs, IFN-λ3. Data are representative of two independent experiments. Paired *t*-test *^/#^*p* < 0.05, **^/*##*^*p* < 0.01, ***p < 0.001 (mean ± SE). *Mock vs. IFN treatments, ^#^M-Mϕ vs. GM-Mϕ.

IFN receptor expression and response to type I and III IFNs was examined in monocytes and Mϕs. Mϕ differentiation increases the abundance of the type I IFN receptor, *IFNAR1* transcript (Figure 2A), and protein (Figure 2B) ~2-fold as compared to monocytes irrespective of stimulus. *IFNLR1* transcript abundance was increased in M-Mϕs and GM-Mϕs over 30- and 60-fold, respectively. The IFN-λ co-receptor IL10RB was also measured, and was not significantly modulated following macrophage differentiation (Figure S5). Consistent with gene expression, IFNLR1 protein was absent in monocytes, and increased in GM-Mϕs compared to M-Mϕs. The IFNLR1 bands at 70 and 45 kDa represent the full length and soluble isoforms of IFNLR1, respectively (24).

To confirm that elevated IFNLR1 expression confers response to IFN-λs, monocytes and Mϕs from three healthy subjects were treated with IFN-λ3 for 15 min and STAT1 phosphorylation (Y701) was examined by Western blot. Monocytes did not phosphorylate STAT1 in response to IFN-λ3, whereas both M-Mϕs and GM-Mϕs were highly sensitive (Figure 2C). All cells demonstrated no STAT1 phosphorylation pre-treatment (Figure S6).

Mϕs were subsequently treated with either interferon-alpha (IFN-α) or IFN-λ3 to determine whether cognate receptor expression defines sensitivity. Following 8 h of IFN-α or IFN-λ3, all measured ISGs were significantly increased by both IFNs (Figure 2D). M-Mϕs were more sensitive to IFN-α, demonstrating stronger induction of all ISGs, particularly *CD80* and *TRAIL*. In contrast, GM-Mϕs were more sensitive to IFN-λ3, increasing the expression of both *ISG15* and *viperin* compared to M-Mϕs. To confirm that macrophage differentiation and not treatment with M- or GM-CSF specifically induce IFNLR1 expression, monocytes were also differentiated using 10% autologous human serum from healthy individuals. Compared monocytes, human serum differentiated macrophages (HS-Mϕs) possess increased *IFNLR1* transcript abundance and possessed similar IFN-λ3 responsiveness as MDDCs and GM-Mϕs, both of which express high levels of *IFNLR1* (Figure S7).

### IFN-λ3 Drives a Pro-inflammatory Macrophage Phenotype

**Figure 3:**
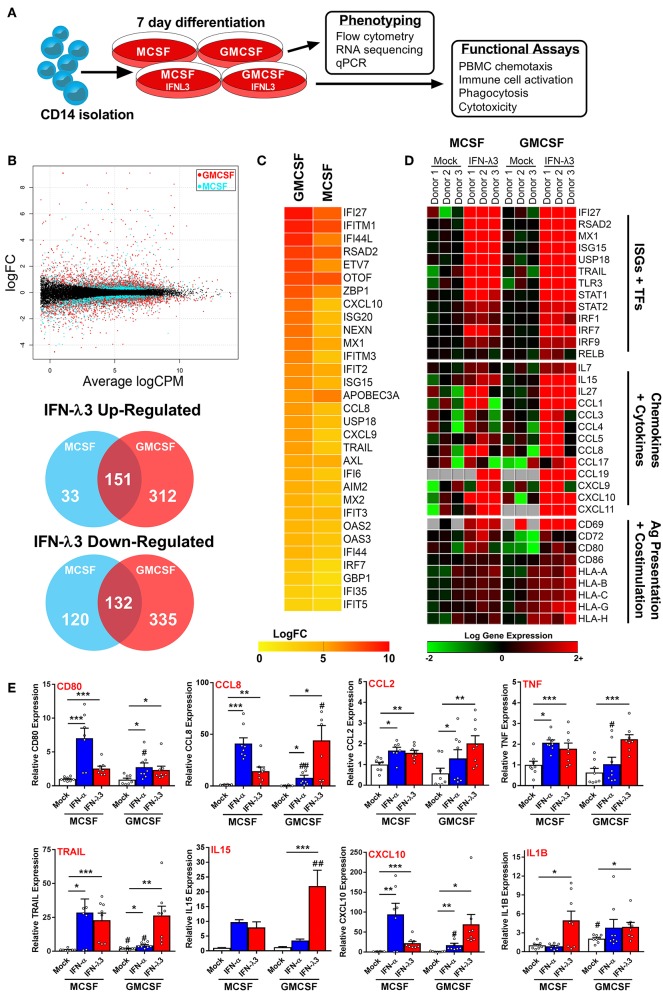
Monocyte differentiation with IFN-λ3 drives a pro-inflammatory macrophage phenotype. Monocytes were cultured with M- or GM-CSF ± IFN-λ3 for 7 days to examine the effect of IFN-λ3 on Mϕ differentiation, followed by phenotypic and functional characterization **(A)**. RNA sequencing (*n* = 3/treatment) demonstrated that GM-CSF Mϕs were significantly more responsive to IFN-λ3, as demonstrated by smear plot and Venn diagram of genes regulated above 2-fold **(B)**. GM-CSF Mϕ ISG induction was significantly stronger, as demonstrated by heat map of gene log 2-fold change (logFC) **(C)**. IFN-λ3 increased transcript abundance of numerous ISGs and transcription factors (TFs) in both Mϕ sets, as well as numerous chemokines, cytokines, and genes responsible for antigen (Ag) presentation and co-stimulation, particularly in GM-CSF Mϕs **(D)**. IFN-λ3 stimulated genes were confirmed by qPCR, using IFN-α differentiated Mϕs as a comparison **(E)** (*n* = 8). Quantitative PCR data are representative of three independent experiments. Paired *t*-test *^/#^*p* < 0.05, **^/*##*^*p* < 0.01, ***p < 0.001 (mean ± SE). *Mock vs. IFN treatments, ^#^M-Mϕ vs. GM-Mϕ.

The robust induction of *IFNLR1* following monocyte plating suggests that monocytes quickly become IFN-λ responsive upon differentiation and transmigration into tissues. Consequently, in the context of chronic antigen exposure, IFN-λ expression at sites of inflammation will likely influence monocyte differentiation and subsequent Mϕ phenotype due to their prolonged exposure throughout the differentiation process. To test this hypothesis, we differentiated monocytes for 7 days with either M-CSF or GM-CSF alone (differentiation stimulus), or in combination with IFN-λ3 (activation stimulus), followed by transcriptional and functional assessment of Mϕ phenotype (Figure 3A).

RNA sequencing of M-Mϕs and GM-Mϕs from three donors was undertaken followed by paired analysis of transformed gene counts (Log 2) between untreated and IFN-λ3 treated Mϕs (Tables S1, S2). The resulting smear plot demonstrates significantly up and down-regulated genes following differentiation of M-Mϕs (blue) and GM-Mϕs (red) with IFN-λ3 (Figure 3B). GM-Mϕs were significantly more responsive to IFN-λ3, up-regulating 463 genes ≥2-fold compared to 184 genes in M-Mϕs. Similarly, GM-Mϕs down-regulated 467 genes ≥2-fold compared to 252 genes in M-Mϕs. IFN-λ driven ISG induction was also collectively higher in GM-Mϕs as demonstrated by the heat map of gene expression LogFC (Figure 3C).

Functional analysis of data from Mϕs differentiated with IFN-λ3 revealed numerous well defined ISGs (e.g., *IFI27, MX1*, and *TLR3*) and transcription factors (STAT and IRF gene families) responsible for ISG gene transcription (Figure 3D). In addition, a Th1 chemokine profile (*CCL3, 4*, and *5* and *CXCL9, 10*, and *11*) responsible for CCR5 and CXCR3 mediated immune cell chemotaxis (39) was found in response to maturation with IFN-λ3, with stronger induction in GM-Mϕs. Up-regulation of immune cell interaction and activation (*CD80, CD86*, and *IL15*) as well as antigen presentation [major histocompatibility complex (MHC) class I HLA genes] was also observed. Using transcriptomic markers of M1 and M2 Mϕ differentiation, IFN-λ3 was found to induce the expression of the majority of M1, but not M2 markers, in both M and GM-differentiated Mϕs, supporting the movement toward an M1 phenotype (GM-CSF *p* < 0.001, M-CSF *p* < 0.05, Sign test null hypothesis of 0.5) (Figure S8).

Gene induction was confirmed by qPCR from a larger group of donors including individuals used for RNA sequencing data, and compared to the effects of IFN-α differentiation. M-Mϕs were considerably more sensitive to IFN-α, whereas GM-Mϕs responded strongly to IFN-λ (Figure 3E). In addition to chemokines identified by RNA sequencing, inflammatory mediators including *CCL2, IL1B*, and *TNF* transcripts were increased by IFN-λ in both Mϕ subsets. To assess the role of differentiation (M- vs. GM-CSF), interferon treatment (IFN-α and -λ3), and their subsequent interaction, a 2-way ANOVA was additionally performed. As expected, all ISGs measured were significantly affected by IFN treatment (*p* < 0.01), however only *CD80* expression was influenced by differentiation (*p* < 0.01). In agreement with RNA-Seq analysis, a significant interaction between IFN treatment and differentiation was observed for all measured genes (CXCL10, CCL8, IL15; *p* < 0.001, CD80; *p* < 0.01, TRAIL, TNF; *p* < 0.05) apart from IL1B and CCL2.

Analysis using Metacore functional annotation software demonstrated that similar networks were activated by IFN-λ3 in both M-Mϕs and GM-Mϕs (Table 1). Immune activation was considerably stronger in GM-Mϕs, with highly significant *p*-values in networks such as antigen presentation and lymphocyte proliferation. Down-regulated gene networks were primarily associated with the cell cycle and protein translation (Table S3). This analysis is consistent with Mϕ BrdU assays, which demonstrated a reduction in BrdU incorporation following IFN-α (*p* < 0.05) and IFN-λ3 (NS) treatment (Figure S9).

**Table 1:** Metacore gene networks up-regulated by IFN-λ3.

**M-CSF+IFN-λ3 Mϕ networks**	***p*-value**
Interferon signaling	9.25E-28
Antigen presentation	2.69E-10
Innate immune response to RNA viral infection	4.08E-08
Inflammasome	1.26E-07
NK cell cytotoxicity	1.26E-06
Chemotaxis	2.72E-06
Lymphocyte proliferation	3.60E-06
IL-10 anti-inflammatory response	4.45E-06
Phagosome in antigen presentation	4.37E-05
IFN-gamma signaling	4.57E-05
**GM-CSF+IFN-λ3 Mϕ networks**	***p*****-value**
Interferon signaling	9.63E-22
Antigen presentation	3.95E-17
Lymphocyte proliferation	7.30E-14
IL-4 signaling	7.17E-10
Innate immune response to RNA viral infection	6.34E-09
NK cell cytotoxicity	1.59E-08
Phagosome in antigen presentation	6.91E-08
Inflammasome	5.37E-07
IFN-gamma signaling	2.07E-07
Leucocyte chemotaxis	2.55E-07

### Interferon Lambda Promotes Lymphocyte Migration and NK Cell Degranulation

**Figure 4:**
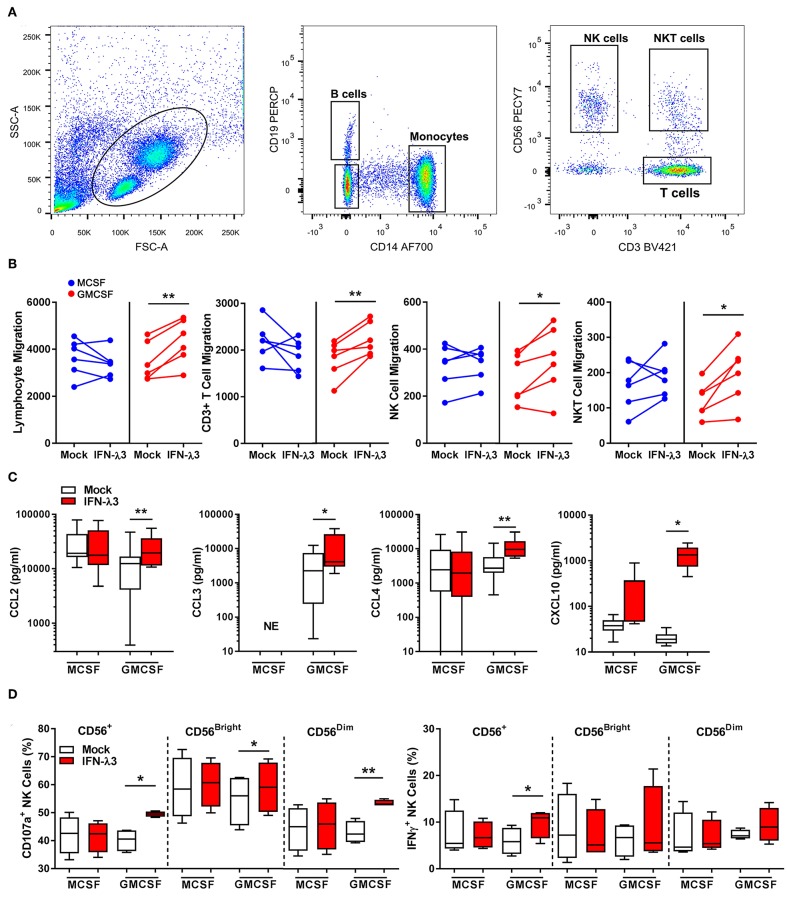
Macrophages differentiated with IFN-λ3 induce lymphocyte chemotaxis and NK cell activation. Transwell inserts containing autologous PBMCs were placed into wells containing 6-day differentiated Mϕs ± IFN-λ3 to examine immune cell chemotaxis (*n* = 6). Migrated cells were analyzed by flow cytometry using CD14, CD19, CD3, and CD56 antibodies to identify the number of migrated monocytes, B, NK, and T cells **(A)**. The addition of IFN-λ3 to GM-CSF Mϕs alone, stimulated lymphocyte migration, with significant increases in NK, NKT, and T cell migration **(B)**. Consistent with migration data, GM-Mϕ CCL2, CCL3,CCL4, and CXCL10 secretion were significantly increased by IFN-λ3, with no significant effect on M-Mϕs **(C)** (*n* = 7). To assess the role of IFN-λ3 on Mϕ activation of NK cells, autologous NK cells were incubated with Mϕs for 16 h a ratio of 1:1, removed and incubated with K562 target cells for a further 6 h to measure cytotoxicity by degranulation and IFN-γ expression (*n* = 4). GM-Mϕs differentiated with IFN-λ3 significantly increased NK cell cytotoxicity as measured by CD107a expression, as well as IFN-γ production **(D)**. Data are representative of two **(B,D)** and three **(C)** independent experiments. Paired *t*-test *p < 0.05, **p < 0.01 (mean ± SE). NE, No expression.

To determine the extent at which Mϕs differentiated with IFN-λ can drive immune cell migration, transwell migration assays were performed using autologous PBMCs. Following 24 h transwell incubation, migrated cells were removed from the lower chamber and analyzed by flow cytometry (Figure 4A). IFN-λ stimulated lymphocyte migration solely in GM-Mϕs by ~25% more than M-Mϕs (Figure 4B), with NK, T, and NKT cell populations being most affected. Separate measurement of GM-Mϕ media demonstrated that IFN-λ3 stimulated a significant increase in CCL2 (1.8×), CCL3 (3×), CCL4 (2.75×), and CXCL10 secretion (~5×), with minimal effect on M-Mϕs (Figure 4C).

Co-culture experiments were next performed to assess the capacity of IFN-λ matured Mϕs to stimulate NK cells *in vitro*. NK cells were incubated with Mϕs overnight, removed, and co-cultured with K562 cells. K562 cells lack MHC class I expression, making them targets for NK cell killing. A significant increase in NK cell degranulation (CD107a), particularly within the CD56^dim^ population, was observed following co-culture with IFN-λ3 treated GM-Mϕs (Figure 4D). NK cell IFN-γ production was also increased following co-culture with IFN-λ3 treated GM-Mϕs, but significance was lost within subgroup analysis. Minimal effect on NK cell function was observed following M-Mϕ co-culture.

### IFN-λ Stimulates Macrophage Phagocytosis and Cytotoxicity

**Figure 5:**
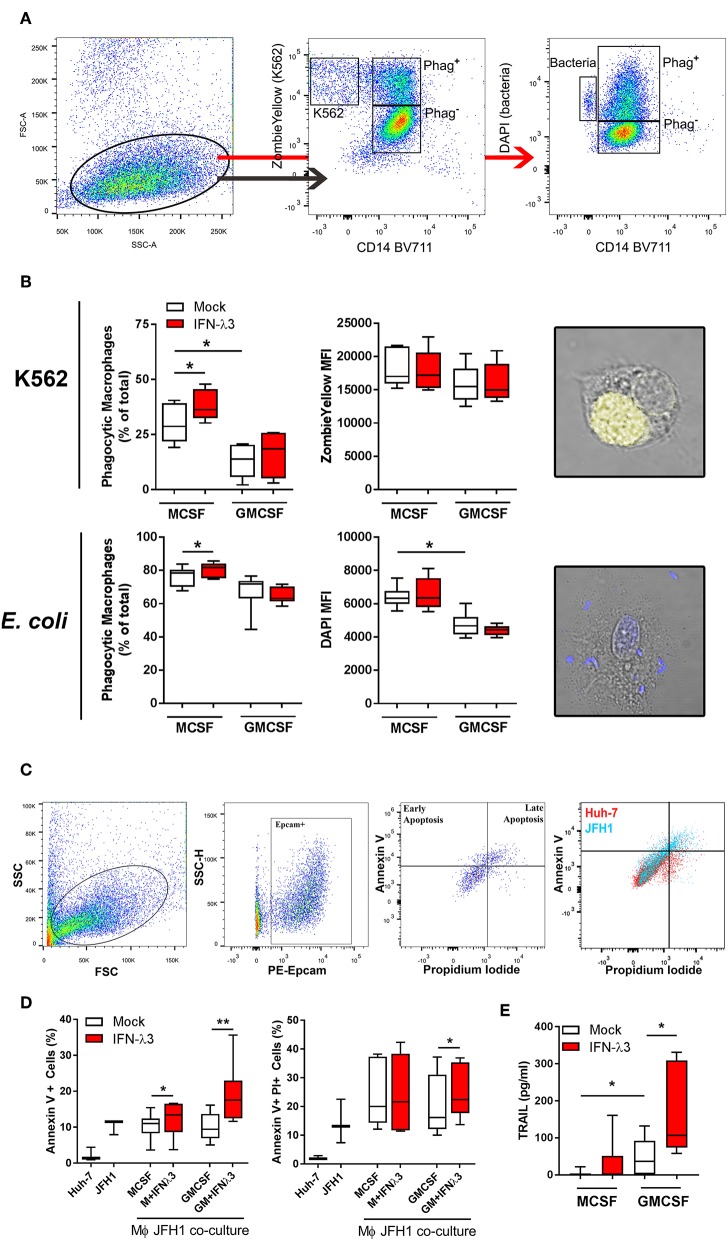
IFN-λ3 stimulates macrophage phagocytosis of apoptotic cells. To examine the role of IFN-λ3 on macrophage phagocytosis, apoptotic K562 cells or *E. coli* were added to Mϕ cultures for 1 h at a ratio of 2:1 and 4:1, respectively. Phagocytic Mϕs were defined as cells double fluorescent for CD14 (BV711), and ZombieYellow viability stain/DAPI, representing target cell engulfment **(A)**. M-Mϕs were more phagocytic than GM-Mϕs toward K562 cells (2-fold increase, *p* < 0.05), a phenotype that was further increased by IFN-λ3 **(B)** (*n* = 6). Similarly, IFN-λ3 increased *E. coli* engulfment in M-Mϕs only. Mϕ MFI, representing the number of cells engulfed remained unchanged in response to IFN-λ3. To assess cytotoxicity toward virus infected cells, Mϕs were co-cultured with JFH1 infected Huh-7 cells, and apoptosis was quantified in Epcam^+^ Huh-7 cells using propidium iodide (PI) and Annexin V **(C)** (*n* = 7). IFN-λ3 stimulated GM-Mϕ cytotoxicity, increasing the percentage of early (Annexin V^+^) and late (Annexin V^+^, PI^+^) apoptotic cells, whereas early apoptosis alone was affected in M-Mϕs **(D)**. TRAIL expression was up-regulated by IFN-λ3 in GM-Mϕs only, providing a possible mechanism of cytotoxicity **(E)**. Data are representative of two independent experiments. Wilcoxon matched pairs signed rank test **(B,E)**, paired *t*-test **(D)** *p < 0.05, **p < 0.01 (mean ± SE).

To examine the effect of IFN-λ3 on Mϕ effector function that is not associated with an inflammatory phenotype, phagocytosis was examined by flow cytometry. Tissue resident Mϕs that demonstrate an M2 phenotype are highly phagocytic and efficient at presenting antigen (40), a phenotype that can be replicated *in vitro* (41, 42). UV induced apoptotic K562 cells stained with Zombie Yellow viability stain or DAPI labeled *E. coli* were incubated with Mϕs for 1 h at a ratio of 2:1 and 4:1, respectively. The ratio of double-fluorescent (phagocytic, CD14+) cells to mono-fluorescent (non-phagocytic, CD14+) cells, as measured by flow cytometry (Figure 5A) was calculated to determine the phagocytic Mϕ percentage (Figure 5B). Confocal microscopy was additionally used to confirm cell engulfment. IFN-λ3 increased phagocytosis of K562 cells (30% increase) and *E. coli* bacteria (10% increase) in M-Mϕ alone. Mϕ MFI, indicating of the number of engulfed target cells, was consistent among populations when K562 cells were used as targets, likely reflecting their large size in comparison to Mϕs. Conversely, M-Mϕ DAPI MFI, representing bacterial engulfment, was significantly higher than M-Mϕs, representing an increase in the number of phagocytosed bacteria. To determine the mechanism by which IFN-λ3 stimulates apoptosis, RNA-Seq data was queried, with a focus on Mϕ receptors responsible for pathogen and apoptotic cell recognition. Consistent with an M2 phenotype, M-Mϕs possessed higher expression of PRRs (*TLR2, TLR4*, and *CD163*), apoptotic ligand receptors (*CD36* and *MERTK*) and complement transcripts (*C1Q* and *C2*) (Figure S10A). IFN-λ3 had minimal effect on the expression of most phagocytic receptors, but significantly increased key members of the complement cascade (C1S and C1R) (Figure S10B). These data suggest that activation of the complement system by IFN-λ3 may stimulate M-Mϕs phagocytosis of both bacterial and apoptotic cells (43, 44), however further functional analysis is required.

To quantify the ability of Mϕs to kill virus infected cells (cytotoxicity), Mϕs were co-cultured with HCV infected (JFH1 strain) Huh-7 cells for 24 h. Following incubation, Huh-7 cells were labeled with Epcam, Annexin V, and propidium iodide (PI) to quantify cells undergoing apoptosis (Figure 5C). Additionally, Huh-7 and JFH1 infected Huh-7 cultures were used as controls to confirm HCV mediated Huh-7 cell apoptosis, as previously described (45). M- and GM-Mϕs differentiated with IFN-λ3 stimulated an increase of early apoptotic (Annexin V+, PI^−^) cells, by ~20 and 90%, respectively, compared to mock treated controls (Figure 5D). GM-Mϕs alone increased Annexin V+, PI+ cells, representing late apoptosis by ~20% following IFN-λ3 treatment. The cytotoxic mechanism by which Mϕs killed infected cells has not been determined, but is likely mediated by soluble factors such as TRAIL that is highly inducible following IFN-λ3 treatment in GM-Mϕs in particular (Figure 5E). Low expression of TRAIL in untreated Mϕs may explain the apparent lack of apoptosis following co-culture. In addition, no nitric oxide production by M- or GM-Mϕs was found in response to IFN-λ3, bacterial or infected cell stimulus. To validate Huh-7 cell apoptosis results, qPCR for apoptosis markers Caspase 3 (*Casp3*), Caspase 7 (*Casp7*), and *Bax* were performed (Figure S11). Co-culture with IFN-λ3 differentiated GM-Mϕs increased *Casp3* and *7* expression by ~6-fold in addition to increasing the antiviral response of Huh-7 cells as demonstrated by strong induction of ISGs *viperin* and *ISG15*.

### Liver Macrophages Are IFN-λ3 Responsive *in vivo*

**Figure 6:**
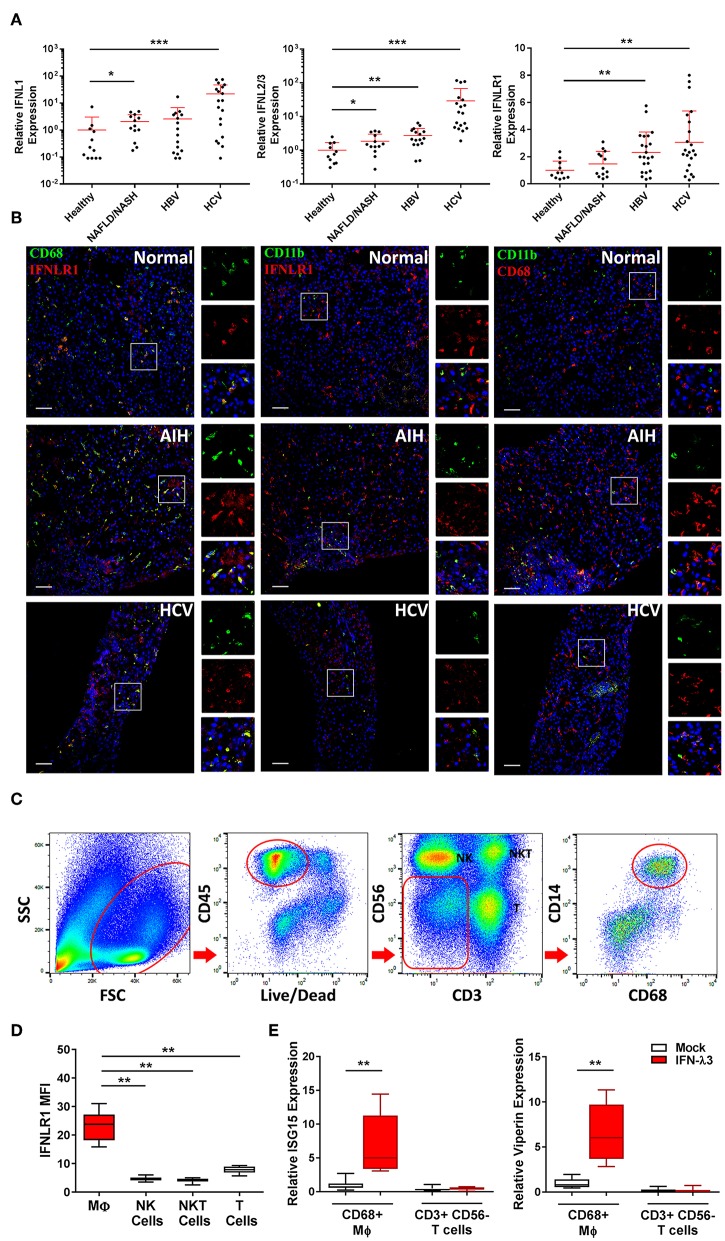
Hepatic IFN-λ3 responsive macrophages are present *in vivo*. *IFNL1, IFNL2/3*, and *IFNLR1* mRNA expression was measured in healthy, NAFLD/NASH, HBV, and HCV infected liver tissue (*n* ≥ 9/group) **(A)**. To examine IFNLR1 expression *in vivo*, biopsy sections obtained from healthy, autoimmune hepatitis, and HCV infected liver tissue were labeled with monocyte/Mϕ markers CD11b and CD68 as well as IFNLR1 antibodies, and examined by confocal microscopy **(B)**. To assess the responsiveness of liver Mϕs to IFN-λ3, immune cells were isolated from fresh liver tissue, sorted by FACS and cultured in the presence of IFN-λ3. Live CD45+ immune cells were sorted based on the expression of CD3 and CD56 into NK and T cell subsets, and CD14 and CD68 into Mϕ subsets **(C)**. IFNLR1 expression, as determined based on IFNLR1 MFI was compared among liver immune cell subsets, and was significantly higher in CD14+, CD68+ liver Mϕs, as compared to NK (CD56+), NKT (CD3+, CD56+), and T cells (CD3+, CD56−) **(D)** (*n* = 6). Sorted T cells and Mϕs were cultured with 100 ng/ml IFN-λ3 for 10 h and ISG mRNA expression was compared to mock treated cells **(E)** (*n* = 8). Scale bars represent 100 μm. Data are representative of one cell sorting experiment. Wilcoxon matched pairs signed rank test, *p < 0.05, **p < 0.01, and ***p < 0.001 (mean ± SE).

To assess IFN-λ production *in vivo*, we measured the expression of *IFNL* genes in liver biopsies of chronic HBV, HCV, and NAFLD/NASH patients and compared them to normal liver tissue from benign liver resections. *IFNL*s were increased in both viral (>10-fold *IFNL1, IFNL2*/3 HCV vs. healthy) and non-viral (e.g., ~2-fold *IFNL1, IFNL2/3* NAFLD/NASH vs. healthy) liver disease (Figure 6A), indicating that chronic inflammatory conditions can increase hepatic IFN-λ expression to facilitate the generation of inflammatory macrophages *in vivo*.

To demonstrate the presence of IFN-λ responsive Mϕs *in vivo*, we performed immunofluorescent labeling of liver tissue from a patient with autoimmune hepatitis (AIH), chronic HCV infection, and normal liver obtained from a cancer resection. Biopsies were labeled with IFNLR1 and CD68 or CD11b antibodies to identify IFN-λ responsive liver Mϕs (Kupffer cells) or myeloid populations (monocytes/macrophages/neutrophils), respectively. As demonstrated in Figure 6B, all CD68+ and a fraction of CD11b+ cells were labeled with IFNLR1.

Immuno-labeling was also performed using CD68 or CD11b in combination with CD3 to demonstrate immune cell proximity in inflamed tissue (Figure S12). CD3+ T cells localized in proximity to CD68 Mϕs, supporting a role for Mϕ derived chemokines as mediators of immune cell trafficking.

To confirm that liver Mϕs are responsive to IFN-λs, CD68+ Mϕs were harvested from liver resection tissue by cell sorting (Figure 6C), and treated with 100 ng/ml IFN-λ3 *ex vivo*. Consistent with our *in vitro* findings, liver Mϕs highly express IFNLR1 compared to liver NK (CD3− and CD56+), NKT (CD3+ and CD56+), and T cells (CD3+ and CD56−) (Figure 6D). In addition, Mϕ IFNLR1 MFI negatively correlated with blood white blood cell count (*r* = −0.678, *p* < 0.05) and positively correlated with hepatic T cell enrichment as a percentage of CD45 cells (*r* = 0.712, *p* < 0.05).

To compare IFN-λ3 sensitivity, T cells and Mϕs from each individual were cultured for 10 h in media alone or with IFN-λ3, followed by quantification of ISG expression. T cells were unresponsive to IFN-λ3 as demonstrated by a lack of *ISG15* and *viperin* induction (Figure 6E). Conversely, IFNLR1 expressing Mϕs were highly responsive to IFN-λ3, increasing the abundance of both transcripts ~6-fold.

## Discussion

**Figure 7:**
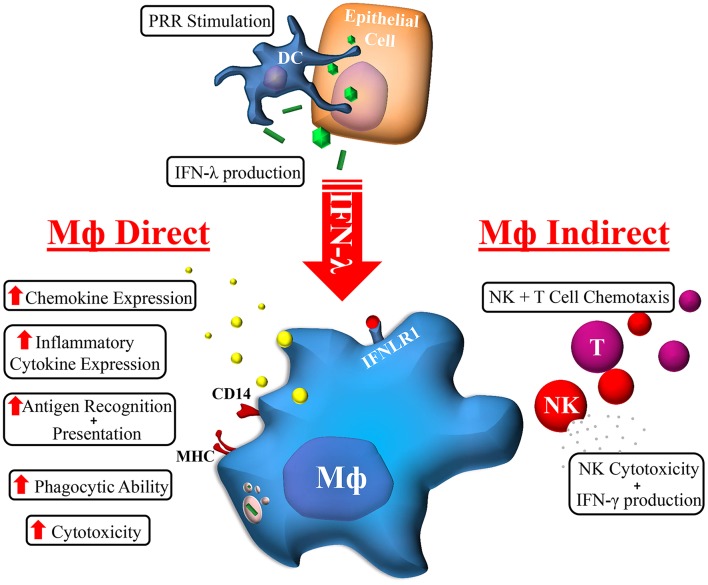
Macrophage orchestration of the IFN-λ immune response. In response to TLR stimulation by viral, bacterial, or fungal pathogens, IFN-λ produced by epithelial (e.g., hepatocytes) or immune cells (e.g., DC) can stimulate the expression of hundreds of Mϕ ISGs. The subsequent effects of IFN-λ on Mϕs can be categorized into direct and indirect effects: Mϕs cytotoxicity and phagocytic ability is increased, as is antigen recognition and presentation, and cytokine/chemokine secretion. Subsequent indirect effects include NK and T cell chemotaxis and increased NK cell cytotoxicity and IFN-γ production.

The cellular and molecular mechanisms by which IFN-λ modulates host responses to viral infections and tissue inflammation remains unclear. Here we undertook comprehensive functional characterization to demonstrate both *in vitro* and *ex vivo*, that macrophages are likely immune cell drivers of IFN-λ mediated hepatic antiviral and inflammatory activities. Unlike monocytes, macrophages are highly sensitive to IFN-λ through the induction of IFNLR1 expression. As such, monocytes likely become IFN-λ responsive upon movement into tissue and subsequent differentiation. Upon IFN-λ stimulation, macrophages develop a robust immune-stimulatory gene signature, expressing hundreds of ISGs, cytokines, chemokines, and co-stimulatory molecules to stimulate both autocrine and paracrine immune cell activation (Figure 7).

IFN-λs are inducible cytokines that drastically increase in abundance upon viral infections, but can also effectively protect against bacterial and fungal insults (46, 47). Activation of TLRs 3, 4, 5, 7, 9 (48, 49) can drive IFN-λ expression, which is dependent factors including cell type and cellular environment. IFN-λs are necessary for epithelial barrier protection in the lungs, liver and gastrointestinal tract, however their dysregulation has been associated with a number of diseases that lack an obvious association with microbial infection. These include chronic inflammatory diseases such as psoriasis (50), systemic lupus erythematosus (51), and asthma (52). Consequently, it is important to understand the direct and indirect molecular mechanisms by which IFN-λs are induced, as well as the responding cellular identities.

The effectiveness of direct acting antiviral therapy for chronic HCV infection has ultimately overshadowed the antiviral role of IFN-λs in the liver, however there remains much to be understood regarding the immuno-stimulatory and potentially destructive roles of these unique cytokines. Recent evidence suggests that *IFNL* genotype influences IFN-λ expression in the liver to facilitate immune cell migration and subsequent inflammation (4, 5). Unlike IFN-λ4 which is weakly secreted by hepatocytes (53, 54), IFN-λs 1–3 are highly expressed, and can exert paracrine effects on surrounding immune cells. This is consistent with reports showing increased Mϕ activation in patients possessing the favorable *IFNL* genotype (55). The importance of the Mϕ response is additionally underscored by the fact that Mϕ but not hepatocyte ISG expression is positively associated with both the favorable *IFNL* genotype that produces increased IFN-λ3 and antiviral response (56, 57).

Both PCR and RNA-Seq analysis support the IFN-λ sensitive nature of GM-Mϕs and highlight the stimulatory role of IFN-λ3. Increased expression of pattern recognition receptors [*TLR3, IFIH1* [MDA5], *DDX58* [RIG-I]] in response to IFN-λ can increase antigen recognition, as we have shown in Figure 5. Numerous pro-inflammatory transcription factors including STATs 1–3, IRFs 1, 7, and 9, AP-1, and NFκB components were activated in response to IFN-λ, as demonstrated by an enrichment of their respective target genes (Table S4). Inflammatory cytokines *TNF* and *IL1B* that are known mediators of hepatocyte apoptosis and liver injury were moderately, albeit significantly induced by IFN-λ treatment alone (Figure 3E), though our data supports additional inflammatory effects of IFN-λ. By strengthening Mϕ recognition and response to pathogen associated molecular patterns, IFN-λs likely sensitize Mϕs to inflammatory stimuli, thus amplifying the strength and/or duration of the inflammatory cascade. This has been demonstrated by Liu et al. who showed that IFN-λ1 can promote IL-12 production in TLR7 stimulated Mϕs (28).

In response to IFN-λ, GM-Mϕs potently express Th1 chemokines including CXCL 9, 10, and 11 as well as IL-15 and IL-27, notable drivers of T and NK cell activation and proliferation. In agreement with RNA-Seq gene expression data, we demonstrated that IFN-λ3 treated GM-Mϕs stimulate T and NK cell chemotaxis and subsequent NK cell cytotoxicity. These data suggest that IFN-λs are strong mediators of the Th1 response and provides a rationale for works by Morrow et al. who showed that IFN-λ3 can increase IFN-γ secretion and degranulation despite T cell insensitivity to IFN-λ (58). A similar phenotype has been observed in tumor model NK cells, where IFN-λ signaling drives NK cell cytotoxicity, suppression of tumor growth and metastases (33, 59). This Th1 skewing effect has been further validated using murine models of Th2 diseases where IFN-λ alleviated symptoms of airway disease (60), intestinal inflammation (61), and conjunctivitis (62).

Interestingly, GM-Mϕs were significantly more responsive to IFN-λ, whereas M-Mϕs where more responsive to IFN-α. These data suggest that while type I and type III IFNs induce a similar gene signature, their respective response is dependent not only on the cell type, but also, the inflammatory phenotype of the responsive cell. This data is consistent with work by Fleetwood et al. that demonstrate a strong dependence on type I IFN signaling in M-CSF over GM-CSF cultured Mϕs (37). Consistent with an M2 phenotype (63), M-Mϕs were not particularly efficient at driving immune cell chemotaxis and activation upon IFN-λ3 stimulation, but were significantly more phagocytic than GM-Mϕs both at baseline and in response to IFN-λ3. These data suggest that IFN-λs are perhaps not inherently inflammatory, but instead promote macrophage effector functions based on location or developmental phenotype.

Our data fills a current gap in knowledge concerning the cellular identities and mechanisms that regulate local IFN-λ mediated inflammation. Because IFN-λ signaling is longer lasting and unlike IFN-α does not become refractory following chronic exposure (64), continuous IFN-λ expression from chronic infections can drive prolonged immune activation. While DCs have a strong IFN-λ response, they are a small population in the liver and migrate toward proximal lymph nodes in response to infection (65). Liver Mϕs (Kupffer cells) on the other hand consist of the ~3/4 of hepatic immune cells, and remain locally to become crucial drivers of localized tissue inflammation (65). Neutrophils are the only other immune cell subset with a defined IFN-λ response, and respond with reduced migration and suppression of inflammation (22, 23, 66).

In summary, we have demonstrated a novel concept whereby Mϕs gain IFN-λ sensitivity quickly following differentiation from monocytes. These data support a pro-inflammatory role for IFN-λs, particularly via recruitment of NK and T cells, chief promoters of inflammation in chronic liver disease. Mϕs bridge the gap between IFN-λ responsive and non-responsive effector cells, and are likely implicated in the elimination of acute infection *and* the promotion of tissue damage in chronic disease.

## Methods

### Patient Cohort

Blood samples were obtained from healthy volunteers at the Westmead Institute of Medical Research. Data points represent individual donors from a cohort of ~20 healthy individuals. Different cohorts of donors were used for individual experiments based on availability. Liver tissue was obtained at Westmead Hospital, Sydney, at the time of needle biopsy [chronic HBV/HCV infection, non-alcoholic fatty liver disease (NAFLD)/NASH, autoimmune hepatitis] or from patients undergoing liver resections (normal tissue). Ethics approval was obtained from the Sydney West Area Health Service and University of Sydney. Informed consent was obtained for all subjects [HREC2002/12/4.9(1564)].

### Immune Cell Isolation, Culture, and IFN Treatment

Peripheral blood mononuclear cells (PBMCs) were isolated from volunteer blood using Ficoll Paque Plus (GE Healthcare). Immune cell isolations were performed using StemCell EasySep Kits, resulting in immune cell purity of >90%. CD14+ monocytes were cultured at 37°C and 5% CO_2_ in RPMI medium containing 10% fetal calf serum (FCS) and 50 ng/ml macrophage colony-stimulating factor (M-CSF, Peprotech) or granulocyte macrophage colony-stimulating factor (GM-CSF, Peprotech) for 7 days, replacing media and removing non-adherent cells at day 4. Mϕ populations were treated with IFN-α (50 U/ml) or IFN-λ3 (100 ng/ml) purchased from R&D Systems. All cytokines were confirmed LPS free.

### RNA Sequencing and Bioinformatics

RNA was extracted using the Favorgen Tissue Total RNA Kit and the sequencing library was prepared using the TruSeq Stranded mRNA Library Prep Kit (Illumina). Single ended RNA sequencing (RNA-Seq) was performed at the Australian Genome Research Facility using the Illumina HiSeq 2500 platform (50 bp read length; minimum of 10^7^ reads per sample). Sequence alignments and gene counts were performed using STAR RNA-Seq aligner version 2.5.1b (67) and paired comparisons were performed using EdgeR version 3.16.2(68). Heat map visualization of RNA-Seq data was performed using GITools (69). Functional analysis of IFN-λ3 mediated gene expression was conducted using Metacore version 6.29 (Thomson Reuters).

### Quantitative PCR

cDNA synthesis was performed using MMLV reverse transcriptase (Promega) and 500 ng of RNA. Gene transcripts were quantified using the Corbett Research Rotorgene 3000 or 6000 thermocyclers with TaqMan primer probes (Applied Biosystems) or custom primers. Quantification of CD80, CXCL10, IFNLR1, and TRAIL were performed using primer probes (Applied Biosystems). Custom primers sets are as follows: CCL2 (CTGCTCATAGCAGCCACCTT, GCACTGAGATCTTCCTATTGGTG), CCL8 (TCCCAAGGAAGCTGTGATCTT, ATGGAATCCCTGACCCATCT), IFNAR1 (TCAGGTGTAGAAGAAAGGATTGAAA, AGACACCAATTTTCCATGACGTA), IFNL1 (AGGGACGCCTTGGAAGAGT, GAAGCCTCAGGTCCCAATTC), IFNL2/3 (GCCACATAGCCCAGTTCAAGTC, GGCATCTTTGGCCCTAAA) IL1B (TCGCCAGTGAAATGATGGCT, GGTCGGAGATTCGTAGCTGG), IL15 (GTGATGTTCACCCCAGTTGC, CATCTCCGGACTCAAGTGAAA), ISG15 (CGCAGATCACCCAGAAGATC, GCCCTTGTTATTCCTCACCA), TNFα (CCCGAGTGACAAGCCTGTAG, TGAGGTACAGGCCCTCTGAT), and viperin (CTTTTGCTGGGAAGCTCTTG, CAGCTGCTGCTTTCTCCTCT). All transcripts were normalized to 18s ribosomal RNA (Applied Biosystems, 4319413E). Standard curves derived from combined assay RNA were used to determine relative expression of genes.

### Digital Droplet PCR (ddPCR)

Immune cell RNA was quantified using the Qubit fluorometer and RNA BR assay kit (Thermo Fisher), and cDNA was synthesized from ≥10 ng of RNA per sample using qScript cDNA supermix (Quantabio). cDNA was combined with ddPCR supermix and droplet generation oil for probes (Bio-Rad), and droplets were generated using the Bio-Rad QX200 Droplet Generator. PCR was performed using IFNLR1 and GAPDH probes according to the manufacturer's instructions, and droplet fluorescence was analyzed using the Bio-Rad QX200 Droplet Reader. Absolute quantification of transcript number was determined using QuantaSoft Analysis Pro software.

### Western Blotting

Mϕs were lysed at 4°C using a denaturing buffer containing protease and phosphatase inhibitors. Protein was quantified and subject to sodium dodecyl sulfate poly-acrylamide gel electrophoresis. Gels were transferred to nitrocellulose membranes, blocked and probed with: IFNAR1 (Abcam, ab45172), IFNLR1 (Sigma Aldrich, HPA017319), STAT1 (Santa Cruz Biotechnology, SC-345), p-STAT1 (Cell Signaling, 9167), and β-actin (Sigma-Aldrich, A1978). Protein bands were visualized on X-ray film using horseradish peroxidase (HRP) conjugated secondary antibodies and the Supersignal West Pico chemiluminescence kit (Pierce Endogen).

### Chemotaxis Assays and ELISAs

Immune cell chemotaxis assays were performed using 1 × 10^6^ autologous PBMCs placed in 5 μM pore size transwell inserts. Assays were performed for 24 h at 37°C and 5% CO_2_. Migrated cells present in the lower chamber were removed by pipetting and characterized by flow cytometry. Zombie Aqua viability stain (BioLegend 423101) and antibodies directed toward CD19 (BioLegend, 302218), CD3 (BioLegend, 300424), CD56 (Becton Dickinson, 335791), and CD14 (Becton Dickinson, 563372) were used to identify immune cell populations. All samples examined by flow cytometry have been treated with Fc block (BD, 564219) prior to staining. ELISAs for CCL2 (R&D Systems, DY279), CCL3 (R&D Systems, DY270), CCL4 (R&D Systems, DY271), CXCL10 (BioLegend, 439904), and TRAIL (Abcam, ab46074) were performed according to the manufacturers' protocols.

### Phagocytosis Assays

To stimulate apoptosis, K562 cells were exposed to UV light for 15 min. Apoptotic K562 cells were stained with ZombieYellow viability stain (Biolegend), after which apoptosis was confirmed with >90% of cells staining positive. Culture media was removed from Mϕ cultures and target cells in RPMI + 10% FCS were added at a ratio of 2:1 (K562). Culture plates were centrifuged at 450 g for 2 min to synchronize phagocytosis. Following 1 h of incubation at 37°C, cells were washed and labeled with BV711 mouse anti-human CD14 antibody (BD Biosciences), and analyzed using the BD Biosciences LSR Fortessa cell analyzer.

### Cytotoxicity Assay

Huh-7 cells were electroporated with JFH1 RNA, a genotype 2 strain of HCV as previously described (70). Upon confirming >85% infection rate by HCV NS5A immunofluorescence, Huh-7 cells were plated in 48 well plates. Day 6 Mϕs were spun down onto Huh-7 cells at 400× g for 5 min at a ratio of 2:1. Following 24 h incubation, macrophages were removed by pipetting, and Huh-7 cells detached using Accutase (Sigma-Aldrich). Huh-7 cells were labeled with Annexin V (Becton Dickinson, 550474), Epcam (Miltenyi Biotec, 130-091253) and propidium iodide (Sigma Aldrich, P4864) in 1× Annexin V binding buffer according to the manufacturers protocol. Cells were washed thoroughly, fixed with 2% paraformaldehyde and analyzed on the BD Biosciences LSR II cell analyzer.

### NK Cell Degranulation and Interferon Gamma Production

Following 6 days of culture, freshly isolated autologous NK cells were added to Mϕs at a ratio of 1:1. Cells were centrifuged at 300 g for 3 min and incubated for 16 h at 37°C with 5% CO_2_. Following stimulation by Mϕs, NK cells were removed by pipetting, and incubated ± K562 cells (1:1 ratio) with an antibody against the degranulation marker CD107a (BD Biosciences 328620), GolgiStop and GolgiPlug transport inhibitor for 6 h at 37°C with 5% CO_2_. NK cell degranulation and interferon gamma (BioLegend, 502509) production was examined by flow cytometry, identifying live NK cell populations using Zombie Aqua viability stain (BioLegend 423101), APC-CY7 CD19 (BioLegend, 302218), Alexafluor 700 CD3 (BioLegend, 300424), PE-CY7 CD56 (Becton Dickinson, 335791), and BUV395 CD16 (Becton Dickinson, 563785).

### Immunofluorescence

Frozen biopsy tissue sections were fixed with acetone, blocked and labeled with primary antibodies against CD11b (Bio-Rad MCA74A488), CD68 (Abcam, AB955), and IFNLR1 (all 1:100 dilution) overnight at 4°C. Secondary fluorescent antibodies (Alexafluor 488/594 anti-rabbit/mouse, Life Technologies, 1:1,000 dilution) and DAPI were applied for 1 h at room temperature, and imaged by confocal microscopy (Olympus FluoView FV1000). The IFNLR1 antibody was validated using siRNA knockdown of IFNLR1 on macrophage cultures, as well as gastrointestinal biopsy tissue to ensure specific labeling (Figure S13).

### Immune Cell Sorting

Liver tissue was diced and incubated for 30 min at 37°C in a dissociation buffer consisting of RPMI medium containing 1 μg/ml DNAse, 0.1 mg/ml Collagenase type IV, and 100 U/ml penicillin/streptomycin. Cells were filtered through a 70 μm cell strainer and centrifuged at 50 g for 5 min to pellet hepatocytes (71). The supernatant containing liver immune cells was washed, pelleted at 400 g and frozen at −80°C until a sufficient number of samples were obtained. Fluorescence-activated cell sorting (FACS) was performed using the Becton Dickinson Influx Cell Sorter using the following panel: Zombie Aqua viability stain (BioLegend, 423102), APC CD45 (BioLegend, 304012), BUV395 CD3 (Becton Dickinson, 563546), PE-CY7 CD56 (Becton Dickinson, 335791), BV711 CD14 (Becton Dickinson, 563372), Alexafluor 488 CD68 (BioLegend, 333812), and PE IFNLR1 (BioLegend, 337804). A cell sort purity of >90% was measured during sorting.

### Statistics

Statistics were performed using GraphPad Prism and were chosen based on the normality of the data, with *p* < 0.05 deemed significant. Student *t*-tests or Mann–Whitney tests were performed on unpaired samples based on data normality. Paired *t*-tests and Wilcoxon matched pairs signed rank test were performed on paired samples based on Gaussian distribution.

## Data Availability Statement

RNA sequencing data has been uploaded into the Figshare data repository: https://doi.org/10.6084/m9.figshare.10324511.v1.

## Ethics Statement

The studies involving human participants were reviewed and approved by Sydney West Area Health Service. The patients/participants provided their written informed consent to participate in this study.

## Author Contributions

SR, ET, MR-M, SS, DB, and GA: designing research studies. SR, RW, ET, and MR-M: conducting experiments. SR, RW, CL, and GA: acquiring and analyzing the data. VL, LY, DB, MD, JG, and GA: providing tissues and reagents and interpreting results. SR, MD, JG, and GA: writing the manuscript.

### Conflict of Interest

The authors declare that the research was conducted in the absence of any commercial or financial relationships that could be construed as a potential conflict of interest.
