# Supplementary material for: Macrophage Coordination of the Interferon Lambda Immune Response
Source: Front Immunol. 2019 Nov 19;10:2674. doi: 10.3389/fimmu.2019.02674 (PMC6878940; doi:10.3389/fimmu.2019.02674)
Supplement: Supplementary file 2 [file Data_Sheet_1.docx]

Supplementary Material

# Supplementary Methods

*Monocyte derived macrophage and dendritic cell culture and phenotyping*

Peripheral blood monocytes were isolated, and differentiated into M- and GM-Mф as in the methods section. In addition, human serum differentiated Mфs (HS-фs) were cultured in 10% autologous human serum, with media change at day 3. Monocyte derived DCs (MDDCs) were generated by culturing monocytes with 50 ng/mL of human GM-CSF and 1000 U/mL of human IL-4 (PeproTech) for 7 days. Culture media was changed on day 3 by spinning down cells, removing half of the supernatant and adding the fresh complete media with a full dose of GM-CSF and IL-4. On day 7, all cells were treated with 5mM EDTA on ice to remove cells, washed with PBS and treated with Zombie Aqua viability stain (BioLegend 423101) and antibodies directed towards CD14 (Becton Dickinson, 563372), CD16 (ThermoFisher, 48-0168-42), and CD1C (BioLegend, 331523). Labelled cells were analysed using the BD Biosciences LSR Fortessa cell analyser.

*Huh-7 cell culture and IFNLR1 knockdown*

Huh-7 cells were cultured in Dulbecco's Modified Eagle's medium (DMEM) containing 10% fetal calf serum and 37°C and 5% CO2. IFNLR1 knockdown was performed using Silencer Select siRNA (ThermoFisher, siRNA ID s46472) and Lipofectamine 2000 (ThermoFisher) according to the manufacturer’s instructions. Briefly, Huh-7 cells were trypsinized, washed in PBS and treated with either 20 nM scrambled (SCR) siRNA, 10, 20 or 50 nM IFNLR1 siRNA upon plating. Cells were cultured with siRNA for 48 h prior to IFNLR1 transcript or protein expression by flow cytometry or Western blot. IFNLR1 expression was validated by Western blot using an additional IFNLR1 antibody (Bioss, BS-11137R).

*Macrophage immunofluorescence and IFNLR1 knockdown*

Monocytes were plated onto glass coverslips and cultured with 50 ng/ml GM-CSF for up to 7 days. Cells were fixed with acetone at day 0 (following monocyte adherence), day 3 and day 7, and were labelled according to methods outlined in the manuscript. Macrophage IFNLR1 knockdown or siRNA control treatment (both 20 nM) were performed as above for 3 days post-monocyte plating, as 7 day treatment resulted in significant cell death.

# Supplementary Figures and Tables

**

**

**Figure S1. GEO dataset monocyte and macrophage IFNLR1 expression.** NCBI GEO datasets were queried for IFNLR1 gene expression array data from monocytes and macrophages. Monocytes were differentiated using M-CSF for 6-7 days (GSE10213, 58310, 37356, 11430, 10220), or human serum for 7 days (GSE22886, 19236) whereas our experimental data utilized GM-CSF for 7 days. Paired t test, * p<0.05, ** p<0.01, *** p<0.001, mean ± standard error.


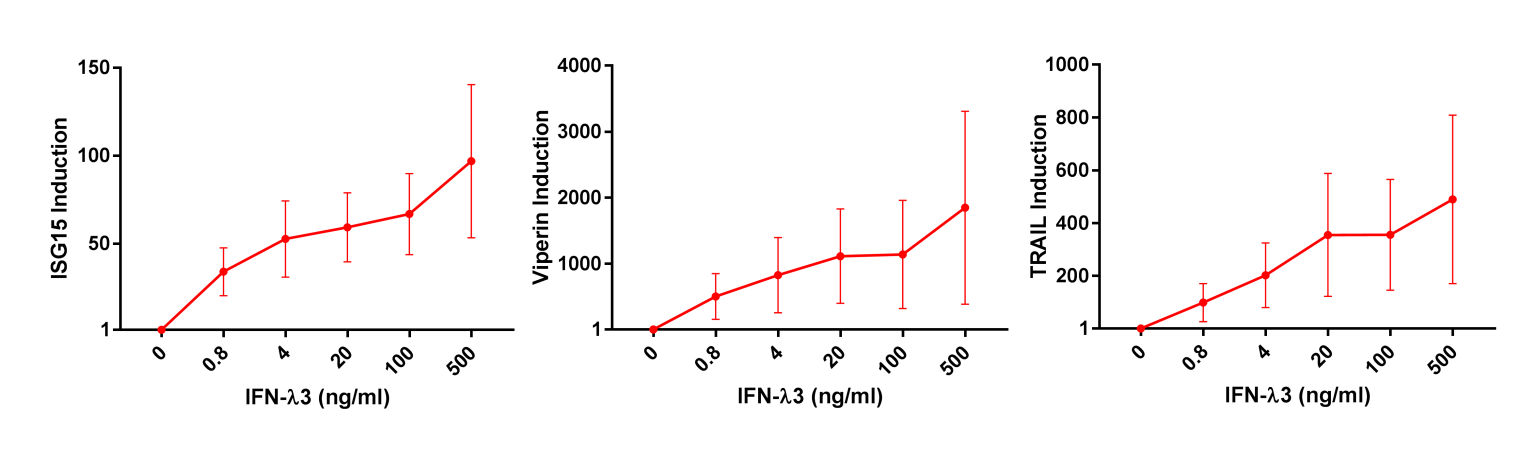


**Figure S2. IFN-λ3 dose-response.** GM-Mфs were treated with different concentrations of IFN-λ3 for 8 h and ISG expression was quantified by qPCR. *ISG15*, *viperin*, and *TNFSF10* (TRAIL) expression increased with increasing IFN-λ3 concentration up to a maximum dose of 500 ng/ml (n=3).

**
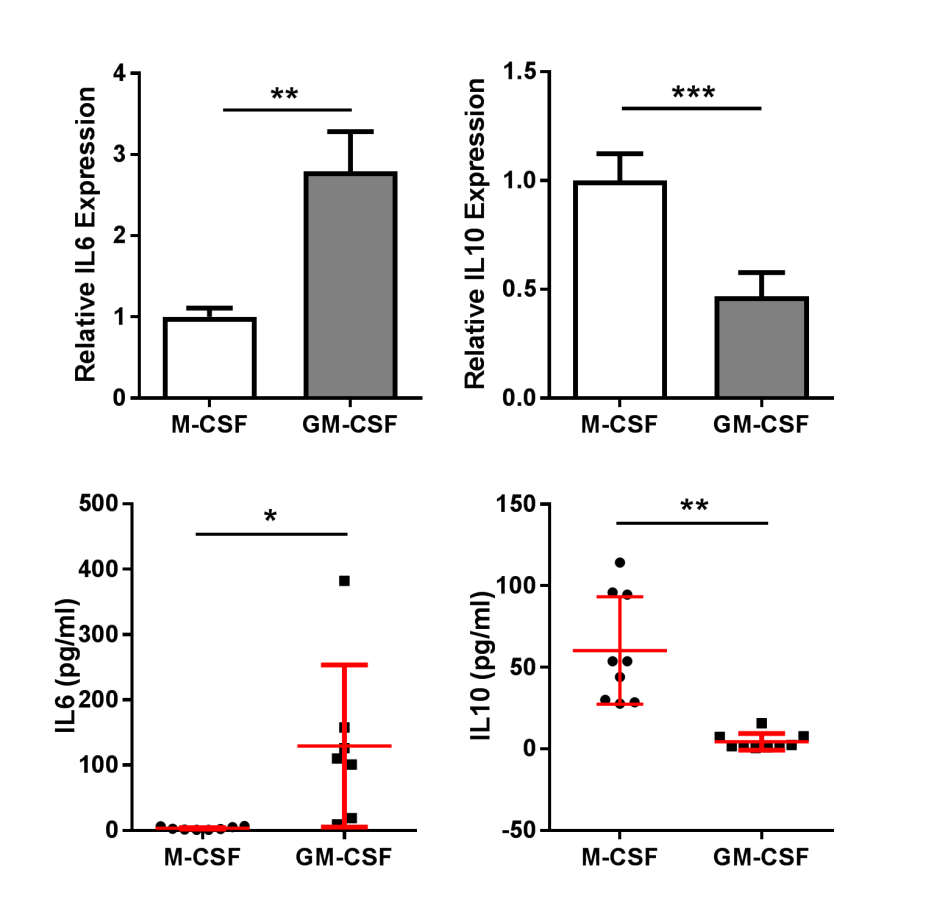
**

**Figure S3. IL-6 and IL-10 expression in M- and GM-CSF differentiated macrophages.** To assess the polarization state of M- and GM-CSF differentiated Mфs, qPCR and bead array was performed for IL-6 and IL-10 representing inflammatory and anti-inflammatory stimuli, respectively. M-CSF differentiated Mфs expressed higher IL-10 while GM-CSF Mфs expressed higher IL-6, as measured by qPCR (n=8) and ELISA (n≥7). Wilcoxon matched-pairs signed rank test, * p<0.05, ** p<0.01, *** p<0.001, mean ± standard error.


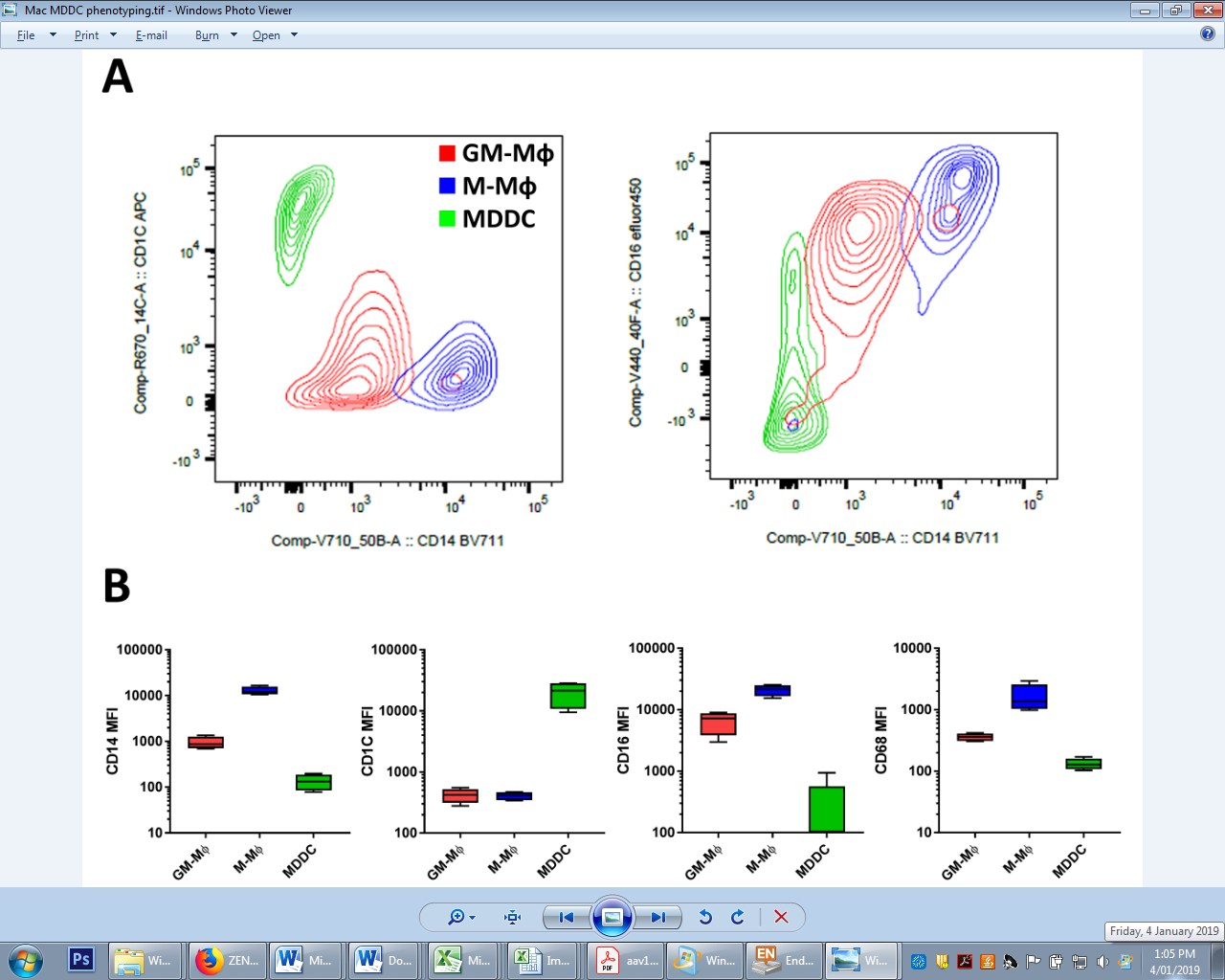


**Figure S4. Monocyte derived macrophage and dendritic cell phenotyping.** To confirm Mф differentiation following M- and GM-CSF, Mф and monocyte derived DC populations were labelled with antibodies directed towards Mф (CD14, CD16), DC (CD1C) and shared (CD68) surface markers and examined by flow cytometry. Contour plots (A) and MFIs (B) demonstrate that Mф populations (single cell, live) possess high surface expression of Mф markers CD14, CD16 and CD68, and low expression of CD1c, indicative of macrophage differentiation. No statistical analysis performed.


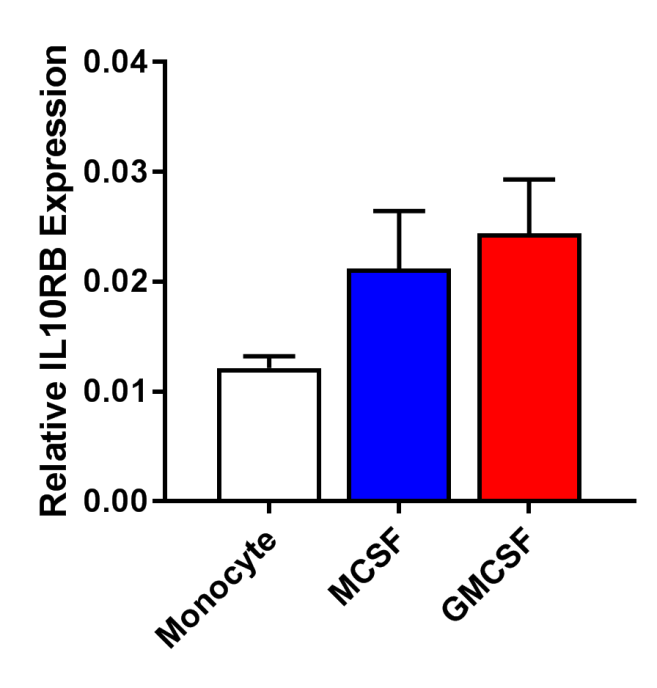


**Figure S5. IL10RB following macrophage differentiation.** *IL10RB* gene expression was measured in monocytes, M-Mфs and GM-Mфs, demonstrating only a modest increase following macrophage differentiation. Unpaired t test, mean ± standard error.


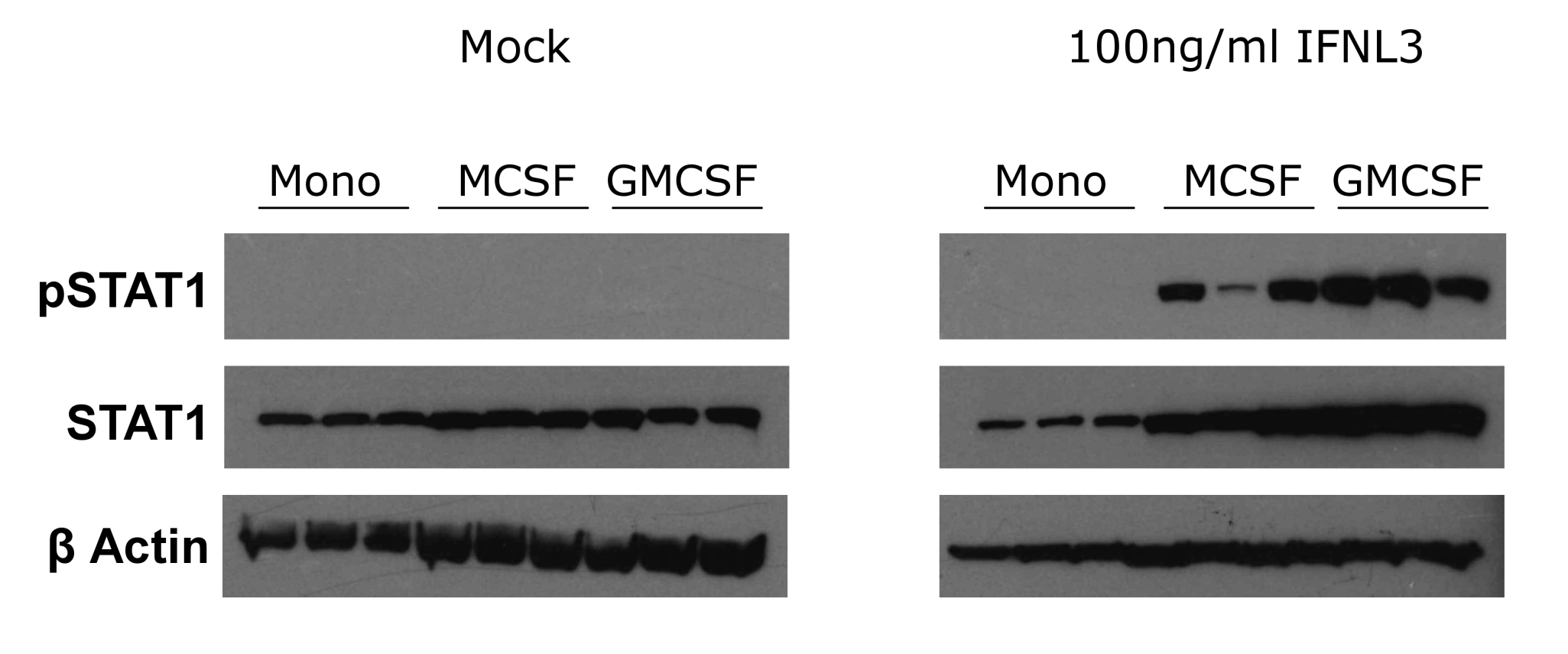


**Figure S6. STAT1 phosphorylation post-differentiation.** Western blot of STAT1 phosphorylation was performed to ensure that differentiation using M- or GM-CSF did not affect baseline STAT activation. No STAT1 phosphorylation (Y701) was measured in either monocyte or Mф populations.


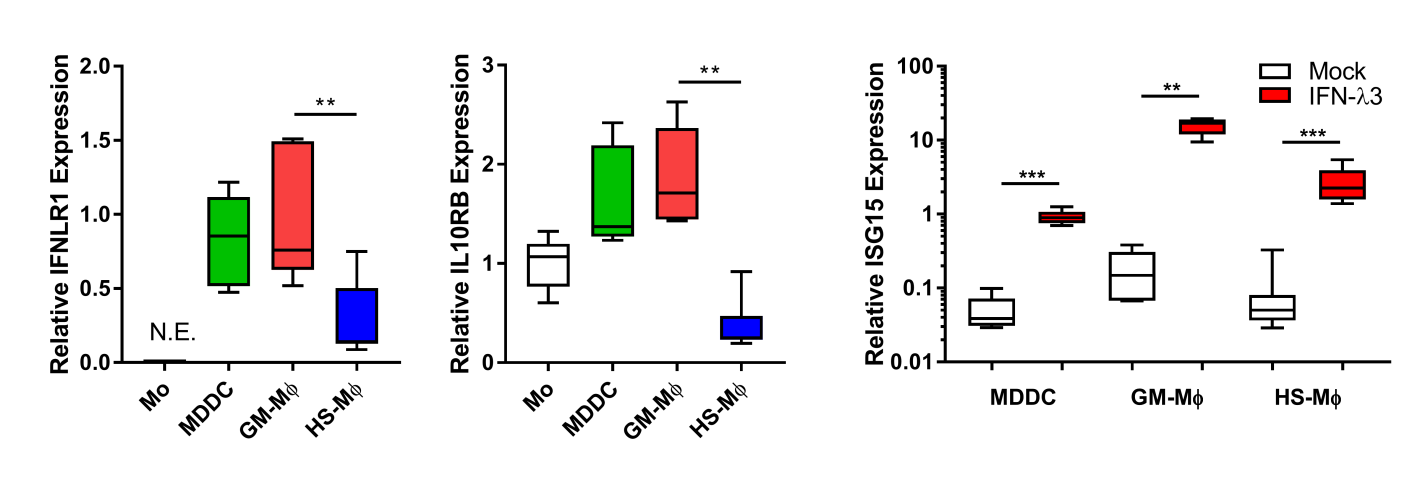

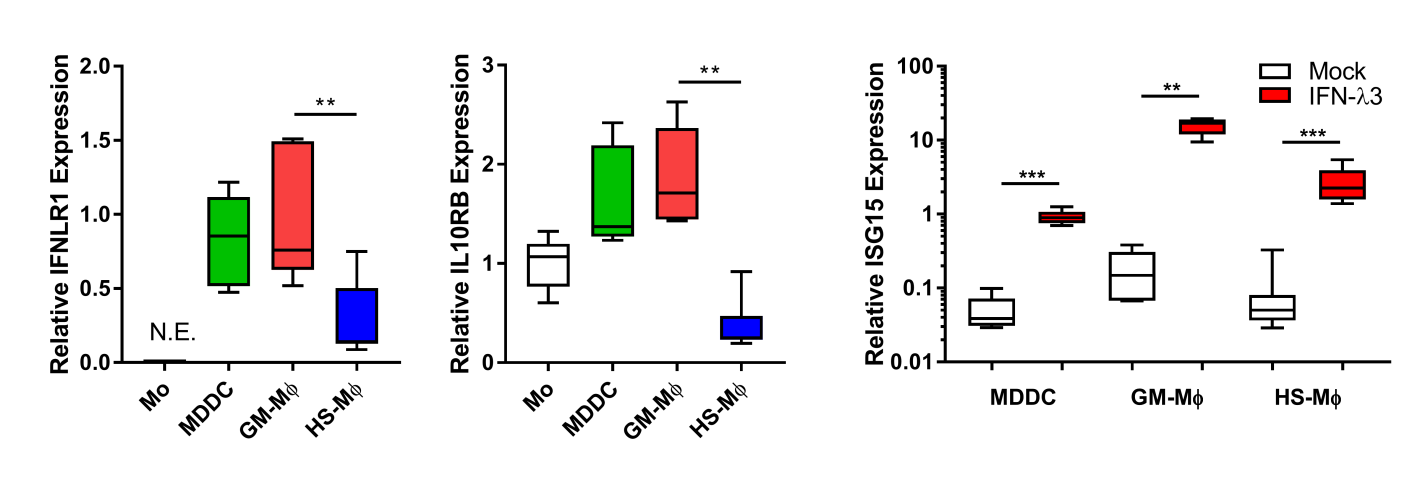


**Figure S7. Human serum differentiated macrophages respond to IFN-λ3.** Compared to monocytes, MDDCs, GM-Mфs and HS-Mфs all up-regulate *IFNLR1*, and develop sensitivity to IFN-λ3 treatment (8 h), as demonstrated by induction of the ISG *ISG15*. Mann-Whitney test, * p<0.05, ** p<0.01, *** p<0.001, mean ± standard error. N.E. not expressed.

**
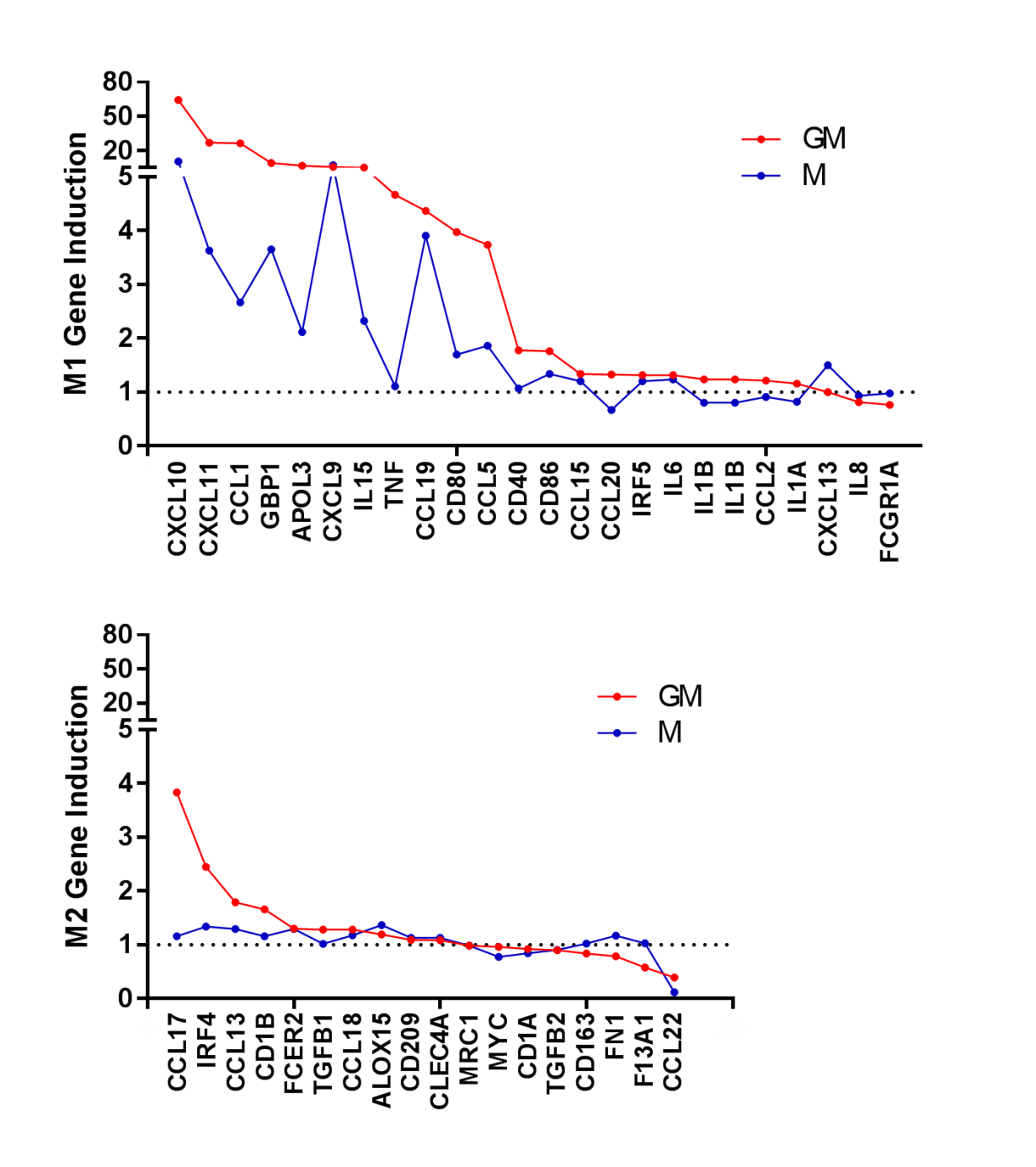
**

**Figure S8. IFN-λ3 up-regulates markers of M1 differentiation.** Following a literature search and identification of transcriptomic M1 and M2 Mф markers (Martinez et al., 2006;Becker et al., 2015;Tarique et al., 2015;Spiller et al., 2016), RNA sequencing results were queried for their induction following IFN-λ3 treatment. Of 24 M1 markers, 22 and 18 were up-regulated by IFN-λ in GM- and M-Mфs, respectively (GM-CSF p<0.001, M-CSF p<0.05, Sign test null hypothesis of 0.5). Of 18 M2 markers, 10 and 13 were up-regulated by IFN-λ in GM- and M-CSF differentiated Mфs, respectively (GM-CSF and M-CSF not significant, Sign test null hypothesis of 0.5).

**
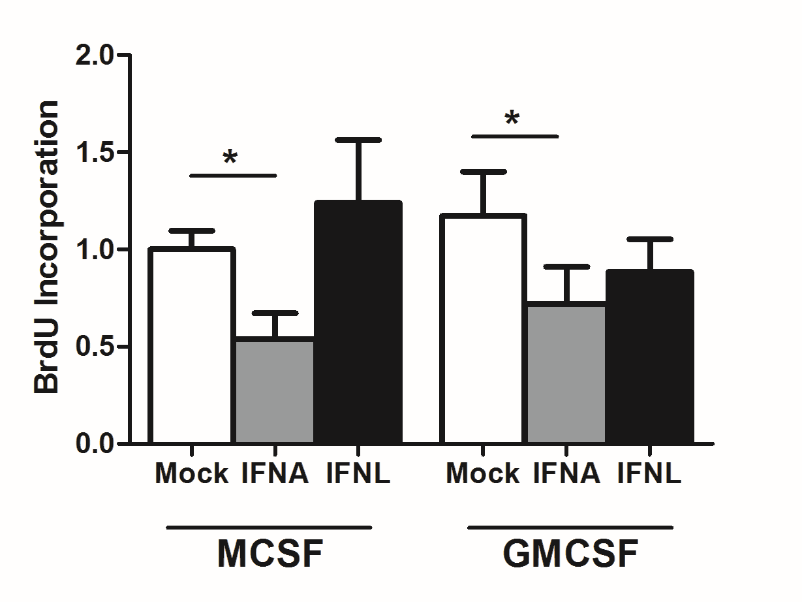
**

**Figure S9. Interferons reduce macrophage proliferation.** Mф proliferation was assayed by BrdU incorporation following 6 days differentiation using either 50 U/ml IFN-α or 100 ng/ml IFN-λ3. IFN-α alone significantly reduced BrdU incorporation following 24 h treatment in both M-CSF and GM-CSF differentiated Mфs (n=6). Wilcoxon matched-pairs signed rank test, * p<0.05, ** p<0.01, *** p<0.001, mean ± standard error.


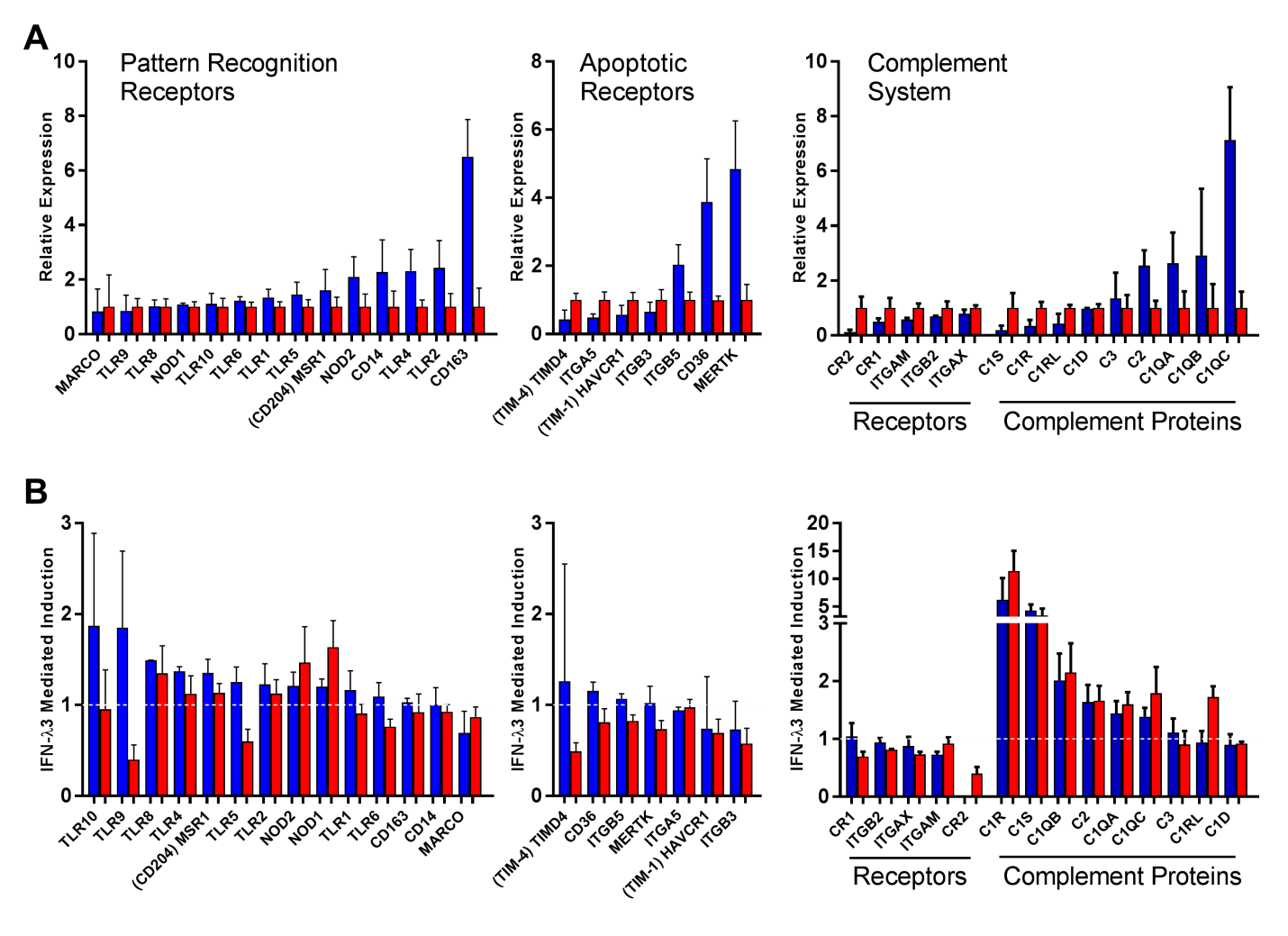


**Figure S10. Phagocytic receptor expression in M- and GM-Mфs.** Transcript expression of phagocytic receptors responsible for recognizing pathogens (pattern recognition receptors), apoptotic cells, and complement opsonized cells were identified in RNA-seq data. Baseline expression (A) of the majority of PRR receptors was elevated in M-Mфs (blue), compared to GM-Mфs (red), whereas only a portion of apoptotic receptors were up-regulated by comparison. Complement receptors were elevated in GM-Mфs, however a number of complement proteins were significantly elevated in M-Mфs. IFN-λ3 induced up-regulation of apoptotic receptors (B), was minimal, however a strong increase in complement factor expression was measured in both Mф populations.

**
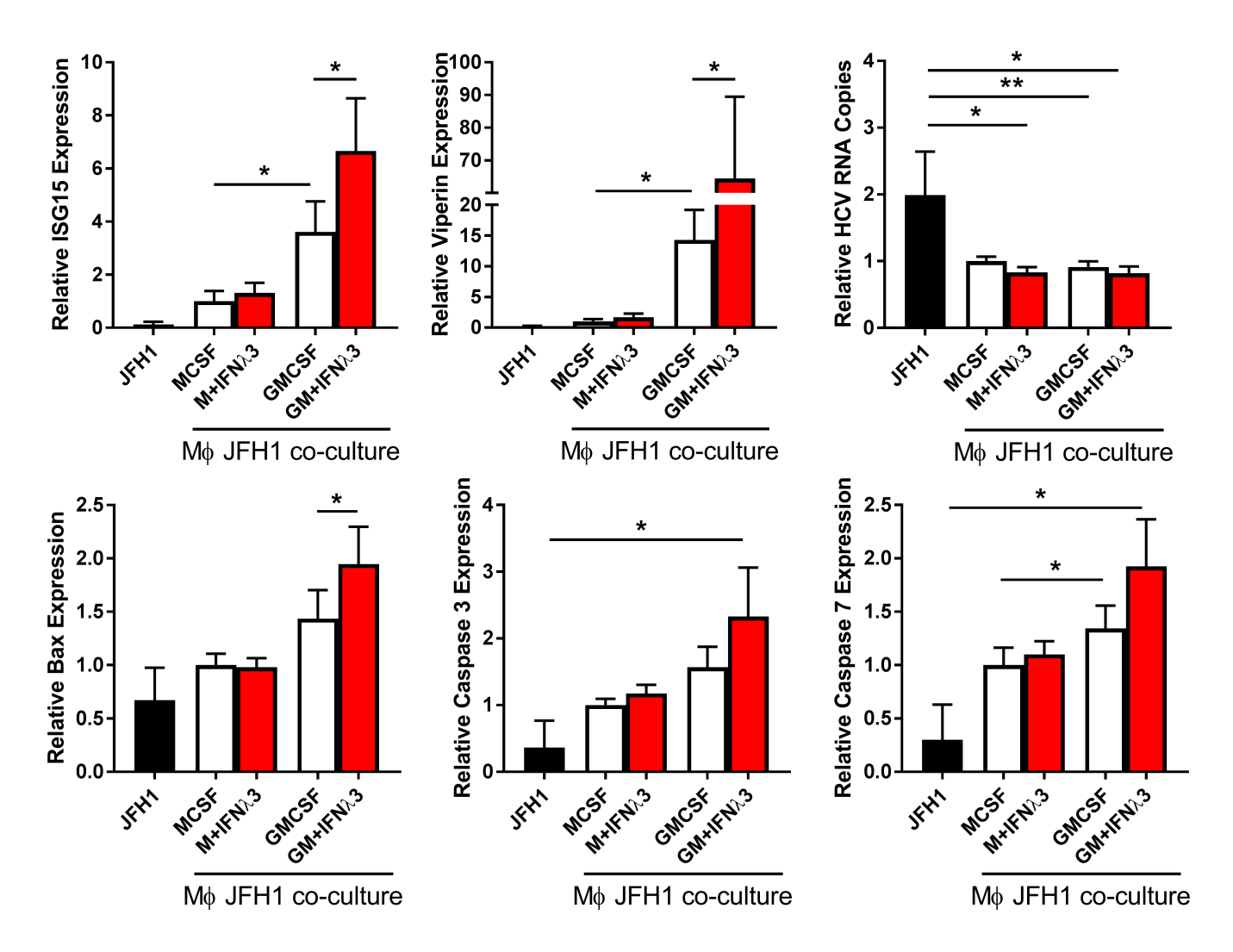
**

**Figure S11. Macrophage co-culture with JFH1 infected Huh-7 cells drives an antiviral and apoptotic response.** Following Mф co-culture, JFH1 infected Huh-7 antiviral and apoptotic gene expression was measured (n≥4). Numerous washes were performed to remove bound Mфs, however minor contamination was almost certainly present. As such, all genes were normalized to the liver specific transcript albumin (Alb). Mф co-culture increased ISGs Viperin and ISG15, while decreasing HCV RNA copies. ISG induction was particularly evident in GM-Mф co-cultures, and was further increased by Mфs differentiated in the presence of IFN-λ3. Compared to JFH1 infected Huh-7 cells, GM-Mф co-culture also induced the expression of apoptotic genes Caspase 3/7 and Bax. Paired t test, * p<0.05, ** p<0.01, mean ± standard error.

**
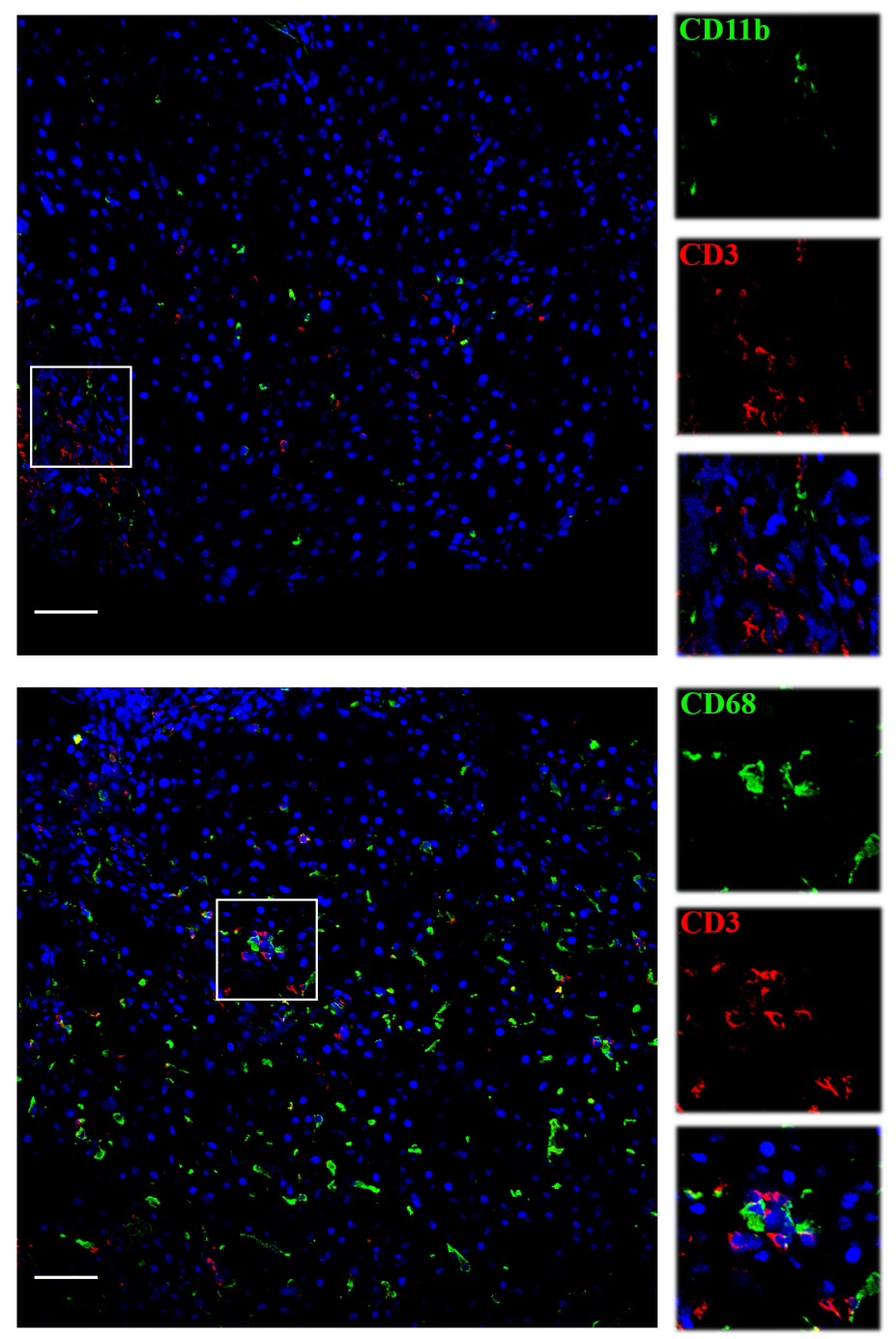
**

**Figure S12. Immunofluorescent labelling of hepatic macrophage: T cell interactions.** A biopsy section from an autoimmune hepatitis patient was labelled with CD3 and either CD11b or CD68 antibodies to identify T cells and macrophages, respectively. Close proximity between T cells and CD68+ Mфs was observed, whereas minimal interaction with CD11b+ cells was found. Scale bars represent 100 µm.

**
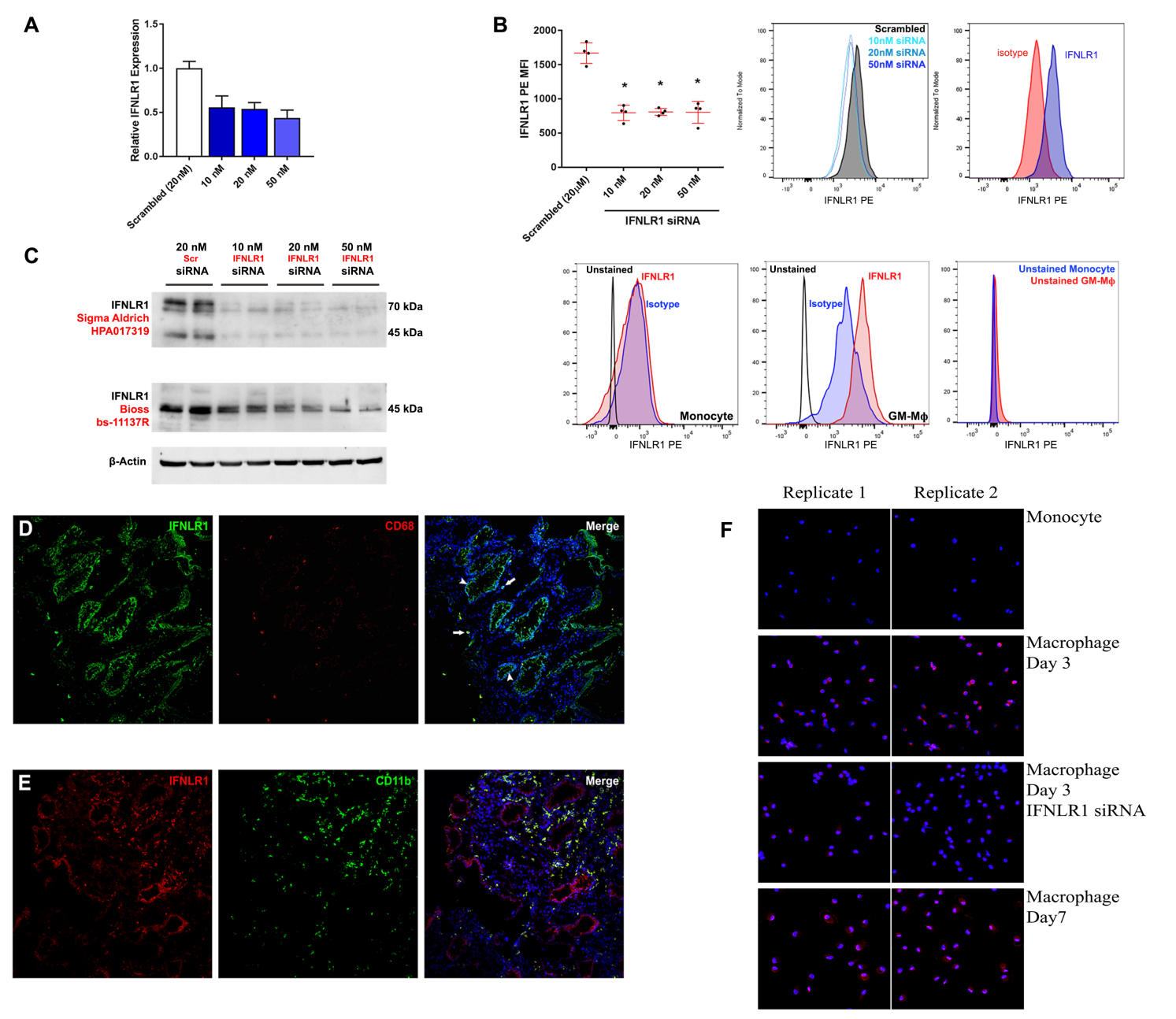
**

**Figure S13. Validation of IFNLR1 antibodies.** IFNLR1 siRNA knockdown was performed on the Huh-7 cell line to validate flow cytometry and Western blot IFNLR1 antibodies. *IFNLR1* gene expression was reduced by approximately 50%, independent of *IFNLR1* siRNA concentration (10-50 nM) (A). As measured by flow cytometry, IFNLR1 MFI was significantly reduced by all siRNA concentrations, when normalized to isotype control (B). In addition, isotype and IFNLR1 MFI was similar in monocytes, however IFNLR1 MFI was approximately twice the value of the isotype control in GM-Mфs. Unstained monocytes demonstrated similar background MFI as GM-Mфs, both of which were <100. Western blot using two separate IFNLR1 antibodies was performed to validate the IFNLR1 antibody (Sigma) in Huh-7 cells following *IFNLR1* knockdown, demonstrating similar results as flow cytometry experiments (C). Notably, the IFNLR1 antibody used in the manuscript (Sigma Aldrich) labelled both 70 and 45 kDa isoforms, whereas the Bioss antibody used to validate the Sigma Aldrich antibody labelled only the 45 kDa isoform. IFNLR1 IF was validated using human colon sections, where cellular architecture is evident, and IFNLR1 expression can be assessed based on anatomical location. IFNLR1 co-localised with CD68 (Mф) (D) and CD11b (myeloid populations) (E), as well as mucosal epithelial cells within colonic crypts that have well documented IFNLR1 expression (Saxena et al., 2017). Lastly, IFNLR1 expression was assessed by IF in monocytes and macrophages ± IFNLR1 siRNA (day 3 only due to toxicity of 7 day siRNA treatment) (F). IFNLR1 labelling was observed in day 3 and day 7 macrophages, but was absent in monocytes and reduced following treatment with 20 nM IFNLR1 siRNA. Unpaired t test, * p<0.05, mean ± standard error.

**Table S3. M-CSF and GM-CSF macrophage gene networks down-regulated by IFN-λ3.**

**
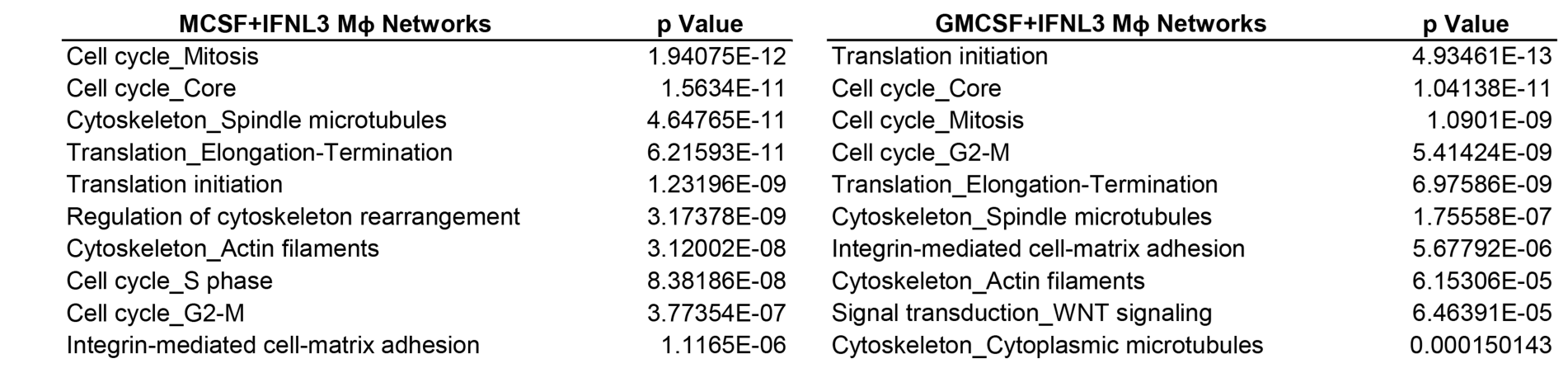
**

**
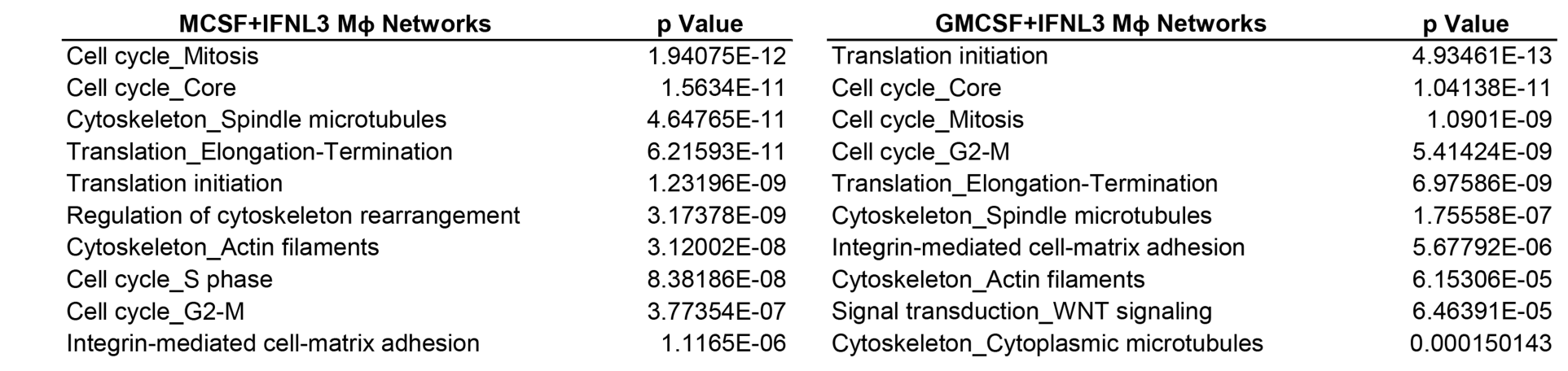
**

**References**

Becker, M., De Bastiani, M.A., Parisi, M.M., Guma, F.T.C.R., Markoski, M.M., Castro, M.a.A., Kaplan, M.H., Barbe-Tuana, F.M., and Klamt, F. (2015). Integrated Transcriptomics Establish Macrophage Polarization Signatures and have Potential Applications for Clinical Health and Disease. *Scientific Reports* 5.

Martinez, F.O., Gordon, S., Locati, M., and Mantovani, A. (2006). Transcriptional profiling of the human monocyte-to-macrophage differentiation and polarization: new molecules and patterns of gene expression. *J Immunol* 177**,** 7303-7311.

Saxena, K., Simon, L.M., Zeng, X.L., Blutt, S.E., Crawford, S.E., Sastri, N.P., Karandikar, U.C., Ajami, N.J., Zachos, N.C., Kovbasnjuk, O., Donowitz, M., Conner, M.E., Shaw, C.A., and Estes, M.K. (2017). A paradox of transcriptional and functional innate interferon responses of human intestinal enteroids to enteric virus infection. *Proc Natl Acad Sci U S A* 114**,** E570-E579.

Spiller, K.L., Wrona, E.A., Romero-Torres, S., Pallotta, I., Graney, P.L., Witherel, C.E., Panicker, L.M., Feldman, R.A., Urbanska, A.M., Santambrogio, L., Vunjak-Novakovic, G., and Freytes, D.O. (2016). Differential gene expression in human, murine, and cell line-derived macrophages upon polarization. *Experimental Cell Research* 347**,** 1-13.

Tarique, A.A., Logan, J., Thomas, E., Holt, P.G., Sly, P.D., and Fantino, E. (2015). Phenotypic, Functional, and Plasticity Features of Classical and Alternatively Activated Human Macrophages. *American Journal of Respiratory Cell and Molecular Biology* 53**,** 676-688.
